# Calcium phosphate coating enhances osteointegration of melt electrowritten scaffold by regulating macrophage polarization

**DOI:** 10.1186/s12951-024-02310-0

**Published:** 2024-01-31

**Authors:** Yubo Shi, Weidong Tao, Wenjing Yang, Lei Wang, Zhennan Qiu, Xiaoli Qu, Jingyi Dang, Jiankang He, Hongbin Fan

**Affiliations:** 1 Department of Orthopedic Surgery, Xijing Hospital, The Fourth Military Medical University, Xi’an, China; 2 Xijing 986 Hospital Department, The Fourth Military Medical University, Xi’an, China; 3 State Key Laboratory for Manufacturing Systems Engineering, Xi’an Jiaotong University, Xi’an, China; 4 Rapid Manufacturing Research Center of Shaanxi Province, Xi’an Jiaotong University, Xi’an, China

**Keywords:** Calcium phosphate, Melt electrowritten, Polycaprolactone, Osteointegration, Macrophage polarization

## Abstract

The osteoimmune microenvironment induced by implants plays a significant role in bone regeneration. It is essential to efficiently and timely switch the macrophage phenotype from M1 to M2 for optimal bone healing. This study examined the impact of a calcium phosphate (CaP) coating on the physiochemical properties of highly ordered polycaprolactone (PCL) scaffolds fabricated using melt electrowritten (MEW). Additionally, it investigated the influence of these scaffolds on macrophage polarization and their immunomodulation on osteogenesis. The results revealed that the CaP coated PCL scaffold exhibited a rougher surface topography and higher hydrophilicity in comparison to the PCL scaffold without coating. Besides, the surface morphology of the coating and the release of Ca^2+^ from the CaP coating were crucial in regulating the transition of macrophages from M1 to M2 phenotypes. They might activate the PI3K/AKT and cAMP-PKA pathways, respectively, to facilitate M2 polarization. In addition, the osteoimmune microenvironment induced by CaP coated PCL could not only enhance the osteogenic differentiation of bone marrow-derived mesenchymal stem cells (BMSCs) in vitro but also promote the bone regeneration in vivo. Taken together, the CaP coating can be employed to control the phenotypic switching of macrophages, thereby creating a beneficial immunomodulatory microenvironment that promotes bone regeneration.

**Graphical abstract:**

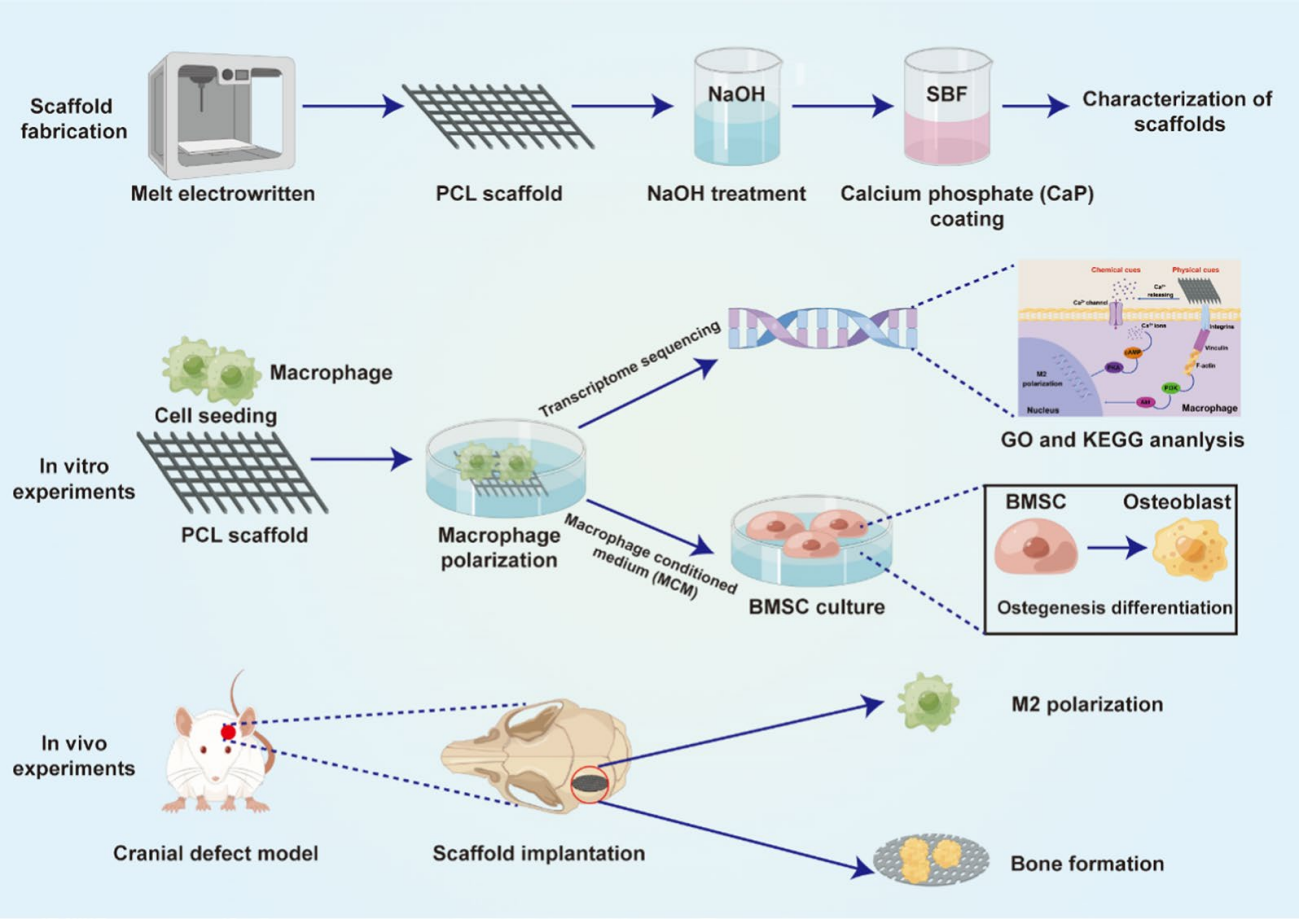

**Supplementary Information:**

The online version contains supplementary material available at 10.1186/s12951-024-02310-0.

## Introduction

In recent decades, researches on biomaterials for bone regeneration have focused on improving the ability of bone-forming cells to generate new bone tissue [1]. However, it is important to recognize that bone regeneration is a complex process that involves immune response, new tissue regeneration, and mature bone formation [2]. When biomaterial scaffolds are implanted, they firstly interact with immune cells and trigger a foreign body response, which may affect subsequent bone healing and remodeling [3]. It has been found that scaffolds which can create an appropriate immune microenvironment may facilitate interactions between host cells and biomaterial, leading to better outcomes [4–6]. Although an immune response is necessary for bone regeneration, an adverse response can cause chronic inflammation and encapsulation, leading to a delayed healing process [7]. Therefore, it is crucial to design scaffolds that can direct the immune microenvironment to promote bone healing.

The primary immune cells drawn to the scaffolds are macrophages. These cells play a crucial role in both foreign body response and bone regeneration [8]. Macrophages are highly adaptable and can be polarized into either classically activated (M1) or alternatively activated (M2) macrophages [9]. M1 macrophages are known for secreting pro-inflammatory factors such as tumor necrosis factor-α (TNF-α), interleukin 6 (IL-6), and interleukin 1 (IL-1). These factors amplify the foreign body response and can lead to a chronic inflammatory response over time [10]. On the other hand, M2 macrophages typically release anti-inflammatory cytokines such as transforming growth factor-β (TGF-β), interleukin 1 (IL-10), and interleukin 13 (1L-13). These cytokines help to suppress inflammation and promote tissue repair and remodeling [11]. Due to their intrinsic plasticity, macrophages can switch between different functional phenotypes in various microenvironments [12]. M1 macrophages can be transformed into M2 when exposed to tissue healing stimuli, while M2 macrophages can be reprogrammed to M1 when exposed to inflammatory signals [13]. Therefore, a promising strategy to enhance the design and development of implanted biomaterial is to induce macrophage polarization towards a regenerative phenotype.

Recent research has shown that the immunomodulatory activities of implanted biomaterials can be modified by changing their surface characteristics (surface wettability or roughness) and surface compositions (inorganic ions or bioactive molecules) [14]. Studies have found that biomaterial surface wettability and roughness can work together to modulate the polarization of primary bone marrow-derived macrophages via Wnt signaling [15]. In addition to surface characteristics, surface compositions also play a crucial role in modulating macrophage polarization. For example, incorporating bioactive molecules like chitosan, heparin, and cytokines into biomaterial surfaces can modulate immunomodulatory activities [16–18]. Recent research has shown that even a small amount of magnesium ions can lead to macrophages adopting a M2 phenotype, which in turn reduces the secretion of inflammatory cytokines [19]. This suggests that it may be possible to fabricate bone implants with immunomodulatory properties by surface modifications.

Previous studies have utilized conventional 3D printing scaffolds for tissue engineering. These scaffolds typically have fiber diameters ranging from 200 to 600 μm [20]. However, due to the large size of the fibers compared to the scale of cells, the scaffolds fail to provide a microenvironment conducive to cell adhesion [21]. Melt electrowritten (MEW) is a 3D printing technology that is gaining popularity due to its ability to create highly ordered structures with a high resolution [22, 23]. This technology can be used to fabricate highly porous scaffolds with appropriate cell adherence, penetration, and proliferation to meet the specific requirements of various tissue regeneration [24–26]. Although MEW scaffolds have been successfully applied in tissue engineering for labrum restoration, periodontal tissue regeneration, and wound healing [27–29], little is known about the application in bone regeneration. Therefore, further studies are needed to explore the potential and efficacy of MEW scaffolds in this area.

Various polymers, such as polyethylene oxide (PEO), poly (L-lactic acid) (PLLA) and polycaprolactone (PCL), have been utilized to fabricate MEW scaffolds [30–32]. Among these polymers, PCL stands out due to its low melting point and excellent printability [33]. However, its inertness and surface hydrophobicity limited application in tissue engineering [34]. To address this issue, researchers have attempted to improve PCL’s surface properties and biocompatibility by coating with bioactive molecules or inorganic ions.

Calcium phosphate (CaP) has a mineral composition similar to that of bone, making it a favorable coating material due to good biocompatibility and osteoinductivity [35, 36]. For instance, CaP coating has been shown to accelerate the osteogenic process of osteoblasts cultured on PCL electrospun scaffolds [37], and a fluorinated CaP coated MEW PCL scaffold has been found to promote osteogenic differentiation of human-derived periodontal ligament stem cells [38]. Although previous studies mainly focused on the osteogenic effect of CaP coating [39, 40], local immune response trigged by scaffolds were rarely studied [41–43]. There is still a lack of conclusive evidence on the osteo-immunomodulatory effect of CaP-coated MEW scaffolds on bone regeneration. Thus, it is worthwhile to investigate the immunomodulatory effects of CaP coated MEW scaffolds. We hypothesize that CaP coating could improve the osteointegration of the MEW scaffold by regulating the immune response.

This study aimed to investigate the effects of CaP coating on the physicochemical properties of MEW PCL scaffolds. The scaffold was characterized by analyzing surface wettability, roughness, and compositions. Furthermore, the modulatory role and underlying mechanism of CaP-coated scaffolds on macrophage polarization and immune microenvironment for osteogenic differentiation was evaluated. Finally, CaP-coated MEW PCL scaffold was implanted to examine the effects of on immune response and bone regeneration.

## Materials and methods

### Scaffold fabrication

The PCL scaffolds were fabricated using MEW. Initially, PCL pellets were heated to 90 ℃ until they melted into a homogeneous fluid. The fluid was then used to print the scaffolds using a 26 G nozzle at a printing speed of 40 mm s^−1^ and a voltage of − 7 kV. During the printing process, the nozzle was positioned 5 mm away from the collector and the air pressure was set at 10 MPa. The CaP coating procedure was performed as follows. Firstly, the scaffolds were immersed in a 2 M NaOH solution for 5 min and washed with Milli Q water five times to neutralize the pH of the rinsing solution. Secondly, the scaffolds were immersed in 1.5 times simulated body fluid (1.5 × SBF) for 48 h at 37 ℃. Finally, the scaffolds were washed with distilled water and vacuum dried. The 1.5 × SBF was prepared following the method described by Kokubo and Takadama [44]. In brief, 0.292 g CaCl_2_, 0.072 g Na_2_SO_4_, 0.311 g MgCl_2_⋅6H_2_O, 0.231 g K_2_HPO_4_⋅3H_2_O, 0.355 g NaHCO_3_, 8.035 g NaCl and 0.255 g KCl were dissolved in RO water at a temperature of 37 °C. The pH of the solution was then adjusted to 7.4 using 1 M HCl. To facilitate subsequent description, the scaffolds were classified as pure PCL scaffolds (PCL), NaOH-treated PCL scaffolds (NaOH-PCL), and CaP-coated PCL scaffolds (CaP-PCL).

### Scaffold characterizations

The surface morphology and element composition were examined by scanning electron microscopy (SEM, Thermo Fisher Scientific, USA) and electron dispersive X-ray diffraction (EDS), respectively. The surface wettability was measured using an automatic contact angle system (JY-82B Kruss DSA), while atomic force microscopy (AFM, Dimension Icon, Bruker, USA) was utilized to examine the surface roughness. X-ray diffraction (XRD, BrukerAXS D8, USA) was utilized to detect the crystalline structures of different scaffolds, and Fourier transform infrared (FTIR, Nicolet Is 10) was used to examine the chemical compositions of different samples. The mechanical properties of the scaffolds were assessed in both tension and compression using the universal mechanical testing machine (CMT6103, MTS). The zeta potential of different scaffold was detected by Zeta Potential Tester (Anton Parr, Austria). The release of calcium ions from the CaP-PCL scaffolds was measured ex vivo using high-resolution inductively coupled plasma mass spectrometry (ICP-MS, Thermo Fisher Scientific, USA). To evaluate the in vitro degradation of various scaffolds, the weight change of each scaffold in Tris–HCl solution was measured at 1, 2, 3, and 4 weeks. This weight change was considered as the degradation rate.

### Cell culture

The macrophage cell line, RAW 264.7, was generously provided by Xijing Hospital. The cell line was cultured in Dulbecco’s Modified Eagle Medium (DMEM, Gibco, USA) supplemented with 10% fetal bovine serum (FBS, Biological Industries, Australia). Primary BMSC were extracted from the femurs and tibias of mouse and cultured in MEM (Gibco, USA) supplemented with 10% FBS.

### Cell proliferation and morphology of macrophage

Before seeding cell, the scaffolds were disinfected with 75% ethanol for 1 h and then washed with PBS to remove residual ethanol. Following this, RAW 264.7 cells were seeded onto scaffolds and incubated. CCK-8 kit was used to evaluate cell proliferation. Briefly, each well was added with 100 µL CCK-8 solution and incubated for 2 h. After that, 100 µL of the mixture was transferred to 96-well plate and the optical density (OD) value was measured using a microplate reader at 540 nm. The morphology of RAW264.7 cells was observed using SEM and confocal laser scanning microscopy (Nikon, Japan). For SEM sample preparation, the cells were fixed with glutaraldehyde, dehydrated with gradient ethanol, and coated with platinum prior to observation. Immunofluorescence staining was performed by fixing the cells with 4% paraformaldehyde and permeabilizing them with Trixon-X. The cytoskeleton and cell nuclei were then stained with phalloidin and DAPI, respectively. Finally, the cells were imaged using confocal microscopy.

### Macrophage polarization in vitro

In this study, RAW264.7 macrophages were seeded onto the scaffolds and incubated for four days. Subsequently, the cells were subjected to immunofluorescence staining, flow cytometry, and western blot assays to detect macrophage polarization. Immunofluorescence staining was performed as described above. In order to identify M1 and M2 macrophages, primary antibodies were employed to detect iNOS and CD86 for M1 macrophages, and CD206 and Arg-1 for M2 macrophages. Subsequently, the cells were subjected to incubation with goat anti-rabbit secondary antibody. After that, the nuclear was counterstained using DAPI solution. Finally, the cells were photographed using confocal microscopy. Flow cytometry assay was performed to detect the surface markers (CD86 and CD206) of macrophage. After incubated for 4 days, RAW264.7 cells on different scaffolds were collected using trypsinization. Subsequently, the cells were subjected to incubation with CD86 and CD206 antibodies, followed by analysis using a flow cytometer. For western blot assay, total proteins extracted from macrophage cells were separated by gel electrophoresis. After transferring the total proteins to polyvinylidene fluoride membranes, they were blocked with 5% milk. Following this, primary antibodies and horseradish peroxidase-conjugated secondary antibodies were incubated with the membranes. Finally, the membranes were photographed using Amersham Imager 600. The details of the antibodies used in this study are listed in Additional file 1: Table S1.

### Quantification of inflammatory response and cytokine release

The study quantified the expressions of anti-inflammatory cytokines IL-4 and IL-10, as well as inflammatory cytokines TNF-α and IL-6 using quantitative real-time polymerase chain reaction (qRT-PCR). Total RNA was obtained by Trizol reagent (Invitrogen, USA) and its concentration was measured with a microplate reader. The cDNA was synthesized using a Reverse Transcription Kit (Takara, Dalian, China) and the amplification process was conducted with the SYBR Taq Kit (Takara, Dalian, China), with GAPDH serving as an internal reference. The premiers are listed in Additional file 1: Table S2. To quantify cytokine release, the medium supernatants were collected after macrophages were cultured on different scaffolds for 4 days. The secretion of cytokines was measured using a commercial cytokine ELISA kit. (Servicebio, Wuhan, China).

### Transcriptome sequencing

After being cultured for 4 days, RAW 264.7 cells were lysed using Trizol reagent (Invitrogen, USA) on different scaffolds. The RNA sequencing was then performed on the resulting lysates using the Illumina NovaSeq 6000. Differentially expressed genes were screened based on the criteria of |log_2_FoldChange|> 1 and *P* < 0.05. The free online platform Novogene was utilized to conduct Kyoto Encyclopedia of Genes and Genomes (KEGG) pathway enrichment analyses. The expressions of key signaling pathways were validated using western blot assay.

### Osteogenic differentiation of BMSC

To explore the impact of macrophage polarization on the osteogenic differentiation of BMSC, supernatants were collected from RAW264.7 cells cultured on various scaffolds. The collected supernatant was centrifuged at × 60 *g* for 5 min and filtered through a 0.22 μm filter to remove any remaining cells. The resulting supernatant was mixed with fresh MEW in a 1:1 ratio to obtain macrophage conditioned medium (MCM). The osteogenic differentiation of BMSC was assessed using various techniques. To detect calcium deposition, the cells were stained with Alizarin Red S (ARS) kit after being incubated for 14 and 21 days with MCM. To quantify the amount of calcium deposition, the cells were added with a solution of 5% sodium 8 dodecyl sulfate (SDS) in 0.5 N HCL for 1 h. The absorbance of this solution at 405 nm was measured using a microplate reader. After a period of 7 and 14 days of incubation, the activity of alkaline phosphatase (ALP) in BMSC was measured using the BCIP/NBT Alkaline Phosphatase Color Development Kit (Beyotime, China). Furthermore, the Alkaline Phosphatase Assay Kit (Beyotime, China) was utilized to determine the ALP activity as per the manufacturer's instructions. The pictures of ARS and ALP staining was captured under phase contrast mode using Carl Zeiss microscopy. Besides, the gene and protein expressions of osteogenesis- related markers (OPN, OCN and RUNX2) were detected using qRT-PCR and western blot as described above.

### Migration of BMSC

The study utilized Transwell and cell scratch healing assays to assess the impact of macrophage conditioned medium (MCM) on the migration of BMSC. In the cell scratch healing assay, BMSC were first seeded into a 6-well plate and labeled with cell tracker. After an incubation period of 24 h, a sterile 200-μl pipette tip was utilized to create a scratch. MCM was added to each well and the healing of scratch was photographed after 0 and 48 h. Transwell assay was performed by seeding BMSC into the upper chamber of the culture medium without FBS, while the lower chamber was added with conditioned medium. After incubation for 24 h, the chamber was washed with PBS, fixed in 4% paraformaldehyde, stained with crystal violet and photographed using a microscopy.

### Animal experiments

The Ethics Committees of The Fourth Military Medical University approved all animal experiments, and all operation procedures were performed in accordance with the National Institutes of Health Guide.

#### Subcutaneous implantation in rats

Each rat was anesthetized with 1% pentobarbital (30 mg/kg) and underwent four surgical incisions on their dorsum. Three types of scaffolds were implanted into subcutaneous pockets, with the fourth incision serving as the surgical control (n = 9 per group). The scaffolds were collected for further studies after 7, 14, and 28 days of implantation.

#### Cranial bone defect model of rats

The rats were anesthetized with 1% pentobarbital (30 mg/kg) and a midline sagittal incision was made to expose their skull. A circular defect of 5 mm diameter was created on both bilateral parietal bones using a circular drill. Subsequently, circular scaffolds measuring 5 mm in diameter and 1 mm in height were placed into the defects, with 6 rats per group. The incisions were then closed and the rats were allowed to recover in their cages. The newly formed bone was detected using Calcein-Alizarin Red staining. After 1 month and 3 months of implantation, the rats were anesthetized and sacrificed for further studies.

#### Micro-CT analysis

The cranial bones were isolated and fixed in 4% paraformaldehyde. Their morphology was subsequently analyzed using Micro-CT, and new bone formation was assessed by calculating bone volume to tissue volume (BV/TV), trabecular thickness (Tb.Th) and trabecular separation (Tb. Sp). To evaluate the in vivo degradability of different scaffolds, the scaffold volume was scanned before and after implantation. The degradation rate was determined by calculating the difference in scaffold volume fractions between the pre- and post-implantation at 1 and 3 months.

#### Histological evaluation

Samples obtained from the subcutaneous regions were treated by fixing them in 4% paraformaldehyde solution and dehydrated using 30% sucrose. The samples were then embedded in optimal cutting temperature (OCT) compound and sliced into 6-μm frozen sections. To evaluate macrophage polarization, HE staining, immunochemistry staining, and immunofluorescence staining were conducted. The cranial bones were divided into two groups: decalcified and undecalcified. The decalcified group underwent decalcification in EDTA for 6 weeks and were then sliced into 6-μm frozen sections for histological staining. The undecalcified group underwent embedding in methyl methacrylate and were sliced into 10 μm sections. Fluorescent-labeled bone sections were imaged using confocal microscopy, and bone formation was assessed via Van Gieson’s staining.

#### Statistical analysis

All data were expressed as average ± standard deviations. The comparison between the groups was performed using a one-way ANOVA. A p-value < 0.05 was considered statistically significant.

## Results and discussion

### Characterization of fabricated scaffolds

**Fig. 1:**
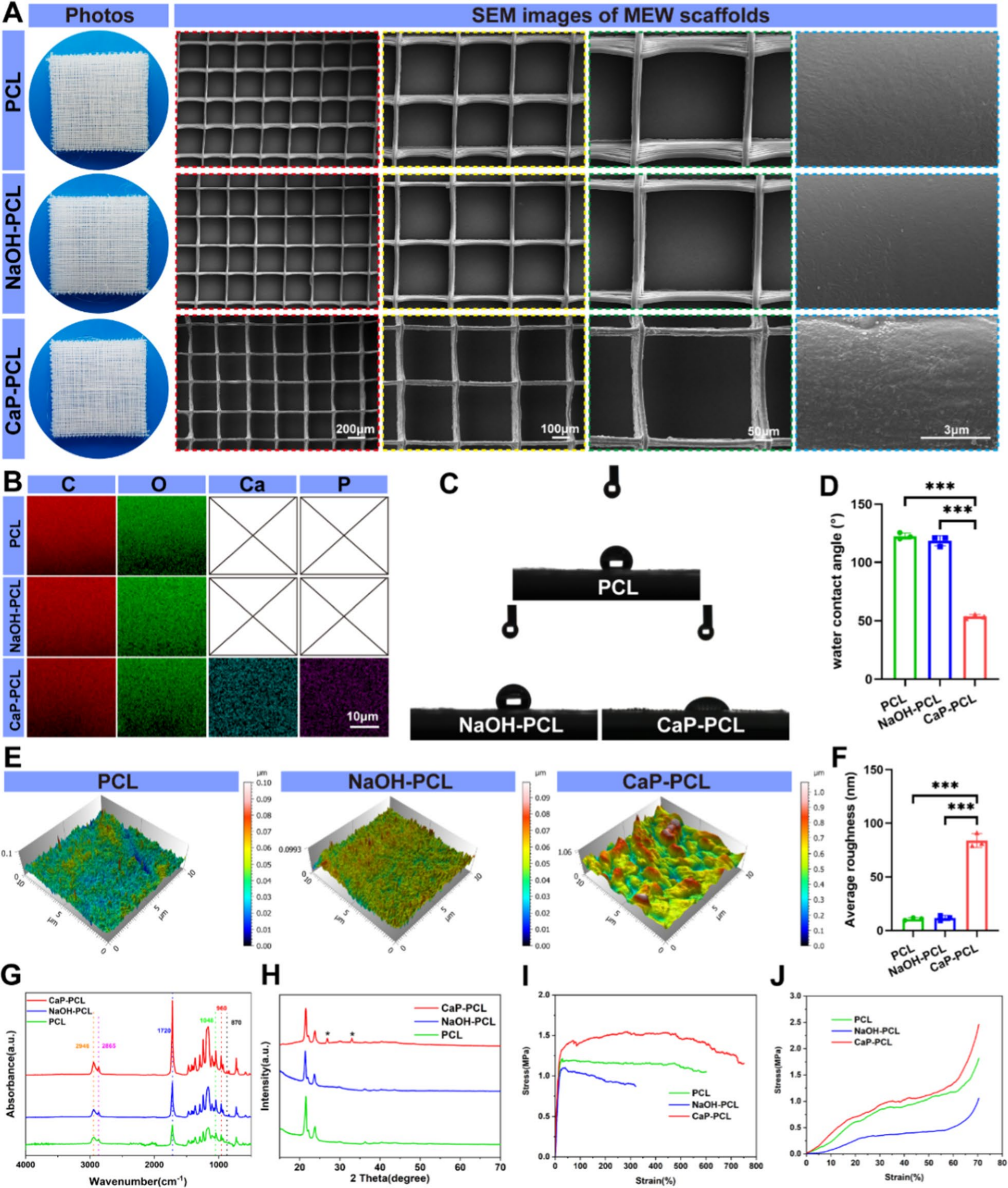
Characterization of fabricated scaffolds. **A** Representative photos and SEM images of different scaffolds. **B** EDS elemental mapping of carbon (C), oxygen (O), calcium (Ca) and phosphorus (P) for different samples. **C**, **D** Water contact angles and the quantification results of different surfaces. **E**, **F** Surface roughness and the quantification results of different scaffolds. **G** FTIR analysis for different scaffolds. **H** XRD analysis for different scaffolds. **I** Tensile stress–strain curves of different scaffolds. **J** Compressive stress–strain curves of different scaffolds. n = 3. ***P < 0.001

The study involved the fabrication of highly ordered PCL scaffolds using MEW. The scaffolds had a uniform 3D architecture comprising of well-aligned fibers (Fig. 1A). The average diameter of the fibers was 20 ± 0.58 μm, while the average spacing between the strands was 300 ± 18.65 μm. Previous studies highlighted the challenge of using PCL in tissue engineering due to its low surface hydrophilicity and inertness [45]. To overcome this limitation, this study proposed the use of CaP coating. However, apatite formation on inert polymers such as PCL requires activation of its surfaces through NaOH treatment, as it does not occur spontaneously [46].

The effect of CaP coating on the surface morphology of PCL was observed using SEM. Upon observation of high-magnification photos, it was found that NaOH treatment did not significantly alter the surface morphology of PCL, except for the presence of scattered nanopits and nanogrooves. However, the CaP-coated scaffolds exhibited a distinct nanostructured surface characterized by round-shaped mineralized nanoparticles (Fig. 1A). Then, elemental compositions of various scaffolds were investigated using EDS. The Ca and P elements were not observed in either PCL or NaOH-PCL scaffolds, but were clearly detected in the CaP-PCL scaffold (Fig. 1B), which had a Ca/P ratio of 1.48 (Additional file 1: Table S3).

It is widely recognized that the surface wettability and roughness of biomaterials have a significant impact on cellular behaviors such as cell proliferation, adhesion, and differentiation [47–49]. Therefore, automatic contact angle system was utilized to evaluate the changes of surface wettability after CaP coating. Results showed that the CaP-coated scaffold had the lowest water contact angle, followed by NaOH-PCL and PCL scaffold (Fig. 1C, D). Likewise, the surface roughness was not significantly affected by NaOH treatment, but it was improved after CaP coating (Fig. 1E, F). In addition, the results zeta potential analysis indicated that both the PCL scaffold and the surface of NaOH-PCL scaffolds had a negative zeta potential − 13.67 ± 0.66 mV and − 14.46 ± 0.61 mV, respectively. However, after CaP coating, the zeta potential decreased to − 16.96 ± 0.69 mV (Additional file 1: Fig. S1).

Subsequently, chemical structures of mineralized particles were measured by FTIR and XRD. The FTIR analysis of PCL revealed characteristic peaks at 2946, 2865, and 1720 cm^–1^, corresponding to the stretching of asymmetric CH_2_, symmetric CH_2_, and C = O bonds, respectively [50]. After CaP coating, peaks indicative of carbonated hydroxyapatite was detected (Fig. 1G). The absorption peak at 870 cm^-1^ was determined to be associated with carbonate (CO2- 3) groups. Additionally, the absorption peaks at 960 cm^−1^ (V_1_) and 1048 cm^−1^ (V_3_) were identified as being correlated to phosphate radical (PO3- 4) groups [51]. Next, XRD analysis was conducted to determine the crystal structure of the CaP coating (Fig. 1H). The results indicated that the PCL exhibited two characteristic peaks at 22.4° and 24.8°, respectively [38]. However, after the CaP coating, new peaks emerged at 26.9° and 32.9°, which were characteristic of carbonated apatite [51, 52]. Taken together, the particles on CaP-PCL scaffolds should be calcium-deficient hydroxyapatite.

In order to assess the mechanical properties of scaffolds, compressive and tensile tests were conducted. The findings from the tensile tests indicated that the tensile strength and tensile Young’s modulus of PCL scaffold were enhanced with the application of CaP coating (Fig. 1I, Additional file 1: Fig. S2). Similarly, the results from the compressive tests showed that the compressive strength and compressive Young’s modulus of PCL scaffold also improved with the CaP coating (Fig. 1J, Additional file 1: Fig. S2). According to the ex vivo calcium release analysis, it was observed that there was a continuous release of Ca^2+^ from day 1 to 28 after soaking the CaP-PCL scaffold in distilled water. However, the slope of the release curve gradually decreased over time, indicating a gradual decrease in the release of Ca^2+^ ions (Additional file 1: Fig. S3). In addition, the results of in vitro degradability analysis showed that the CaP-PCL scaffold had a higher degradation rate compared to the PCL and NaOH-PCL scaffolds after being soaked in Tris–HCl solution for 4 weeks (Additional file 1: Fig. S4). This could be attributed to the CaP-PCL scaffold’s higher hydrophilicity, which allowed for more solution infiltration and consequently faster degradation. Taken together, CaP coating proved to be a straightforward and efficient method for enhancing the physicochemical characteristics of PCL scaffold.

### Cell proliferation and morphology of macrophage

**Fig. 2:**
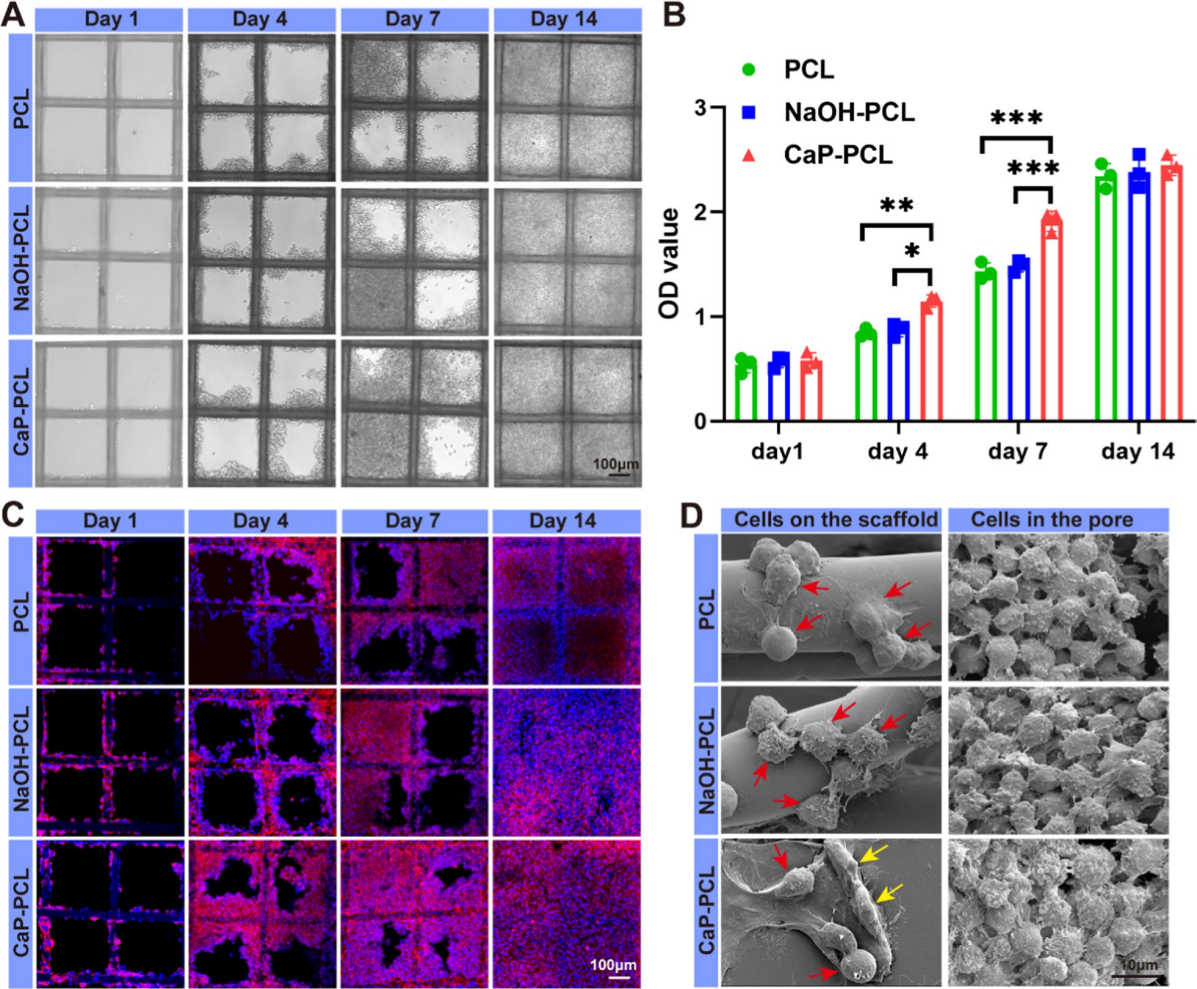
Cell proliferation and morphology of macrophage. **A** The optical images of macrophages on different scaffolds from day 1 to day 14. **B** CCK-8 assay was performed to evaluate cell proliferation of macrophages. **C** Confocal microscopy images of macrophages cultured on different scaffolds from day 1 to day 14. **D** Representative SEM images of macrophages on the scaffold and in the pores (red arrow: pancake-like shape, yellow arrow: elongated shape). n = 3. *P < 0.05, **P < 0.01 and ***P < 0.001

From a cellular compatibility standpoint, it was observed that RAW 264.7 cells demonstrated a gradual increase in proliferation across all scaffolds from day 1 to day 14 (Fig. 2A). According to CCK-8 results, RAW264.7 cells exhibited a higher proliferation rate on CaP-PCL scaffolds compared to the other two scaffolds from day 1 through 7. However, there was no significant difference in proliferation rate among the three groups on day 14 (Fig. 2B). The phenomenon might be attributed to the fact that appropriate surface hydrophilicity and roughness can facilitate cell proliferation in the initial stage [53, 54]. However, once the pores of the scaffolds were filled with cells, no additional space was available for further cell proliferation.

Next, the morphology of RAW264.7 cells was observed using confocal microscopy and SEM. During the initial stage, only a limited number of cells adhered to the fiber surface. However, from day 4 to 7, the cells proliferated and formed cell clusters in certain areas of the fiber surface. As the available fiber surfaces became occupied, the cells started filling up the empty square cavity, resulting in the formation of a cell cluster (Fig. 2C). In addition, SEM analysis revealed interesting findings regarding the morphology changes of RAW264.7 cells. The results indicated that the morphology of cells adhered to the surface of different scaffolds varied. Specifically, RAW264.7 cells on the CaP-PCL scaffold exhibited an elongated shape, while those on the PCL scaffold took on a pancake-like form. However, RAW264.7 cells within the pores of the scaffolds exhibited similar morphology and were not attached to any external support. They exhibited a spheroid morphology and were completely surrounded by neighboring cells, suggesting that they were growing in a three-dimensional (3D) manner (Fig. 2D). The phenomenon might be attributed to the fact that cells displayed different shapes in both the 2D and 3D growth patterns [55]. It seemed that RAW264.7 cells adhered to the scaffold grew in a 2D manner because the scaffold provided a surface for cell attachment and the subsequent protrusion of pseudopodia in various configurations. On the other hand, RAW264.7 cells within the pores grew in a 3D manner due to the absence of attachment sites, resulting in cells adopting similar shapes. Together, these results indicated that the CaP-PCL scaffold exhibited favorable cytocompatibility.

### Macrophage phenotypic switching in vitro

**Fig. 3:**
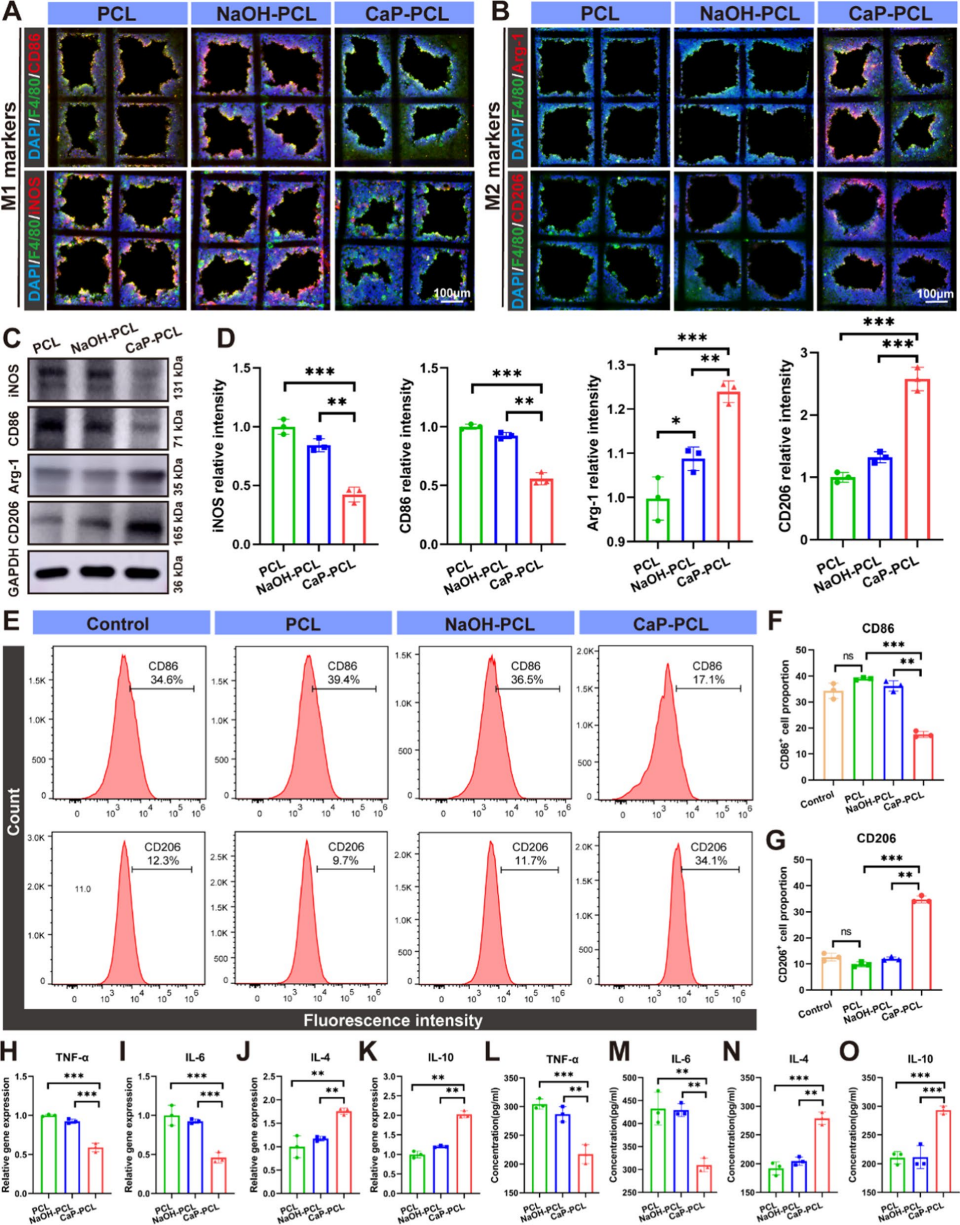
Macrophage phenotypic switching in vitro. **A**, **B** Immunofluorescent images of macrophages on different scaffolds: green (F4/80, a monoclonal antibody specifically directing against the mouse macrophage), red (M1 marker: CD86 or iNOS and M2 marker: CD206 and Arg-1). **C**, **D** The protein expressions and quantification results of macrophage-related markers. **E** Flow cytometry results of CD86 and CD206 expression of macrophages. **F**, **G** The quantification results of flow cytometry. **H**–**K** The mRNA expression of pro-inflammatory genes (TNF-α and IL-6) and anti-inflammatory genes (IL-4 and IL-10). **L**–**O** ELISA results of cytokines TNF-α, IL-6, IL-4 and IL-10. n = 3. **P < 0.01 and ***P < 0.001

To examine the impact of CaP coating on the modulation of macrophage polarization, immunofluorescence staining was employed to detect M1 surface markers (CD86 and iNOS) and M2 surface markers (CD206 and Arg-1) in RAW 264.7 cells [56, 57]. The study revealed that there was no significant difference in macrophage polarization between PCL and NaOH-PCL scaffolds. However, the CaP-PCL scaffold exhibited the lowest fluorescence intensity of CD86 and iNOS positive cells, while demonstrating the highest fluorescence intensity of CD206 and Arg-1 positive cells (Fig. 3A, B). Likewise, the western blot analysis revealed that the expression of iNOS and CD86 were suppressed, whereas the expression of Arg-1 and CD206 were upregulated in macrophages that were cultured on CaP-PCL scaffold when compared to the other two scaffolds (Fig. 3C, D). Subsequently, flow cytometry analysis was conducted to further validate the macrophage polarization on different scaffolds. The results indicated that the CaP-PCL scaffold exhibited the highest proportion of M2 macrophages and the lowest proportion of M1 macrophages (Fig. 3E–G).

Meanwhile, the macrophage-related genes expressions and cytokine secretion were detected by qRT-PCR and ELISA assay. The results of the qRT-PCR analysis demonstrated that the macrophages seeded on the CaP-PCL scaffold exhibited significantly reduced expression levels of M1 related genes (TNF-α and IL-6) and increased expression levels of M2-macrophage related genes (IL-4 and IL-10) when compared to the PCL and NaOH-PCL scaffolds (Fig. 3H–K). Additionally, the ELISA assay yielded similar results. Specifically, the secretion levels exhibited a decrease in pro-inflammatory cytokines such as TNF-α and IL-6, along with an increase in anti-inflammatory cytokines such as IL-4 and IL-10, upon application of the CaP coating (Fig. 3L–O). Taken together, these results indicated that the application of CaP coating would be more effective in promoting the phenotypic switch of macrophages from M1 to M2.

### Immunomodulation-enhanced osteogenic differentiation of BMSC in vitro

**Fig. 4:**
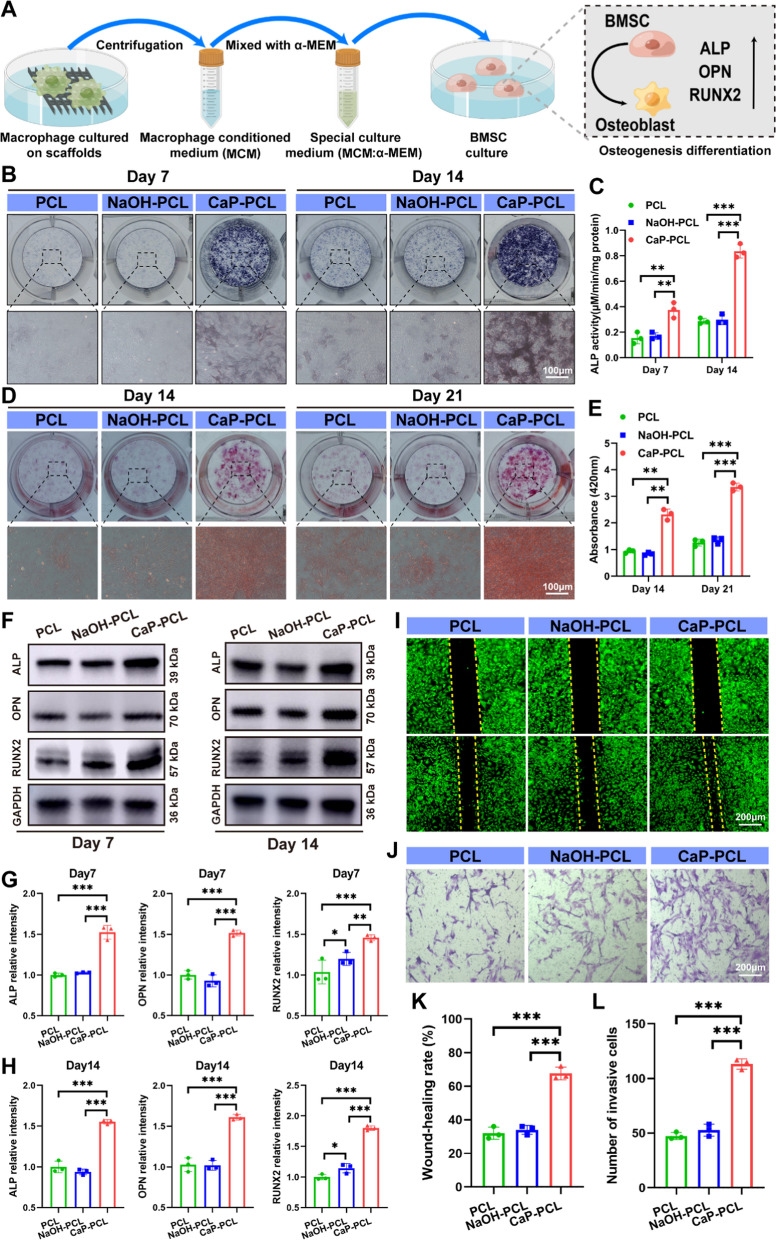
Immunomodulation-enhanced osteogenic differentiation of BMSC in vitro. **A** The illustration of experimental design. **B**, **C** ALP staining of BMSCs and the quantification results. **D**, **E** ARS staining of BMSCs and the quantification results. **F**, **G**, **H** The protein expressions of osteogenic differentiation markers and their quantification results. **I**, **K** The wound-healing assay of BMSC and the quantification results. **J**, **L** The Transwell assay of BMSC and the quantification results. n = 3. **P < 0.01 and ***P < 0.001

After implantation, BMSCs and immune cells were recruited to the surface of the bone implant [58]. The immune microenvironment surrounding the implants played a crucial role in regulating the osteogenic differentiation of BMSCs [59, 60]. Accordingly, the impact of macrophage immune response on in vitro osteogenic differentiation was studied through the use of macrophage conditioned medium (MCM) (Fig. 4A). After being cultured for 7 and 14 days, ALP staining revealed that the MCM obtained from CaP-PCL scaffold exhibited significantly deeper staining compared to the PCL and NaOH-PCL scaffolds (Fig. 4B). This observation was further supported by quantitative analysis of ALP activity (Fig. 4C). Similarly, the results of Alizarin Red S (ARS) staining for calcium deposition at day 14 and 21 indicated that the CaP-PCL scaffolds had larger and more numbers of mineral nodules compared to the PCL and NaOH-PCL scaffolds (Fig. 4D, E). In order to determine the expression levels of osteogenic differentiation markers, namely osteopontin (OPN), Runt-related transcription factor 2 (RUNX2) and ALP, qRT-PCR and western blot were employed to detect their corresponding mRNA and protein expressions. As expected, the mRNA and protein expressions of osteogenic differentiation markers were found to be significantly higher in the CaP-PCL group compared to the other two groups on day 7 and 14 of culture (Fig. 4F–H, Additional file 1: Fig. S5A, B). Meanwhile, the results were also confirmed by immunofluorescence images, which showed that the CaP-PCL derived MCM had the ability to enhance the osteogenic differentiation (Additional file 1: Fig. S5C-F). In addition, the migratory abilities of BMSC were found to be enhanced when treated with CaP-PCL derived MCM, as compared to other groups (Fig. 4I–L). To further investigate the osteoinductive and osteoimmunomodulatory effects of different scaffolds, two types of culture conditions were employed for BMSCs (Additional file 1: Fig. S6A). In the first condition (I), BMSCs were directly seeded on the different scaffolds without the presence of macrophage-conditioned medium (MCM), aiming to explore the osteoinductive effect induced by the CaP coating of the scaffolds. In the second condition (II), BMSCs were seeded on different scaffolds along with the addition of MCM, mimicking the in vivo situation. This allowed for the evaluation of both the osteoinductive and osteoimmunomodulatory effects. After being culture for 14 days, ALP staining demonstrated that BMSCs cultured on the CaP-PCL scaffold exhibited more intense staining compared to the PCL and NaOH-PCL scaffolds. Moreover, BMSCs seeded on the CaP-PCL scaffold with the addition of MCM displayed even deeper staining compared to the CaP-PCL scaffolds without MCM (Additional file 1: Fig. S6B, D). Furthermore, the findings from Alizarin Red S (ARS) staining (Additional file 1: Fig. S6C, E) and western blot analysis (Additional file 1: Fig. S6F) were in line with the results obtained from ALP staining. Despite the direct promotion of osteogenesis from CaP coating, these results indicated that CaP coating mediated macrophage regulation, which had an impact on the osteogenic differentiation of BMSCs.

### Mechanism analysis of CaP-PCL induced macrophage polarization

**Fig. 5:**
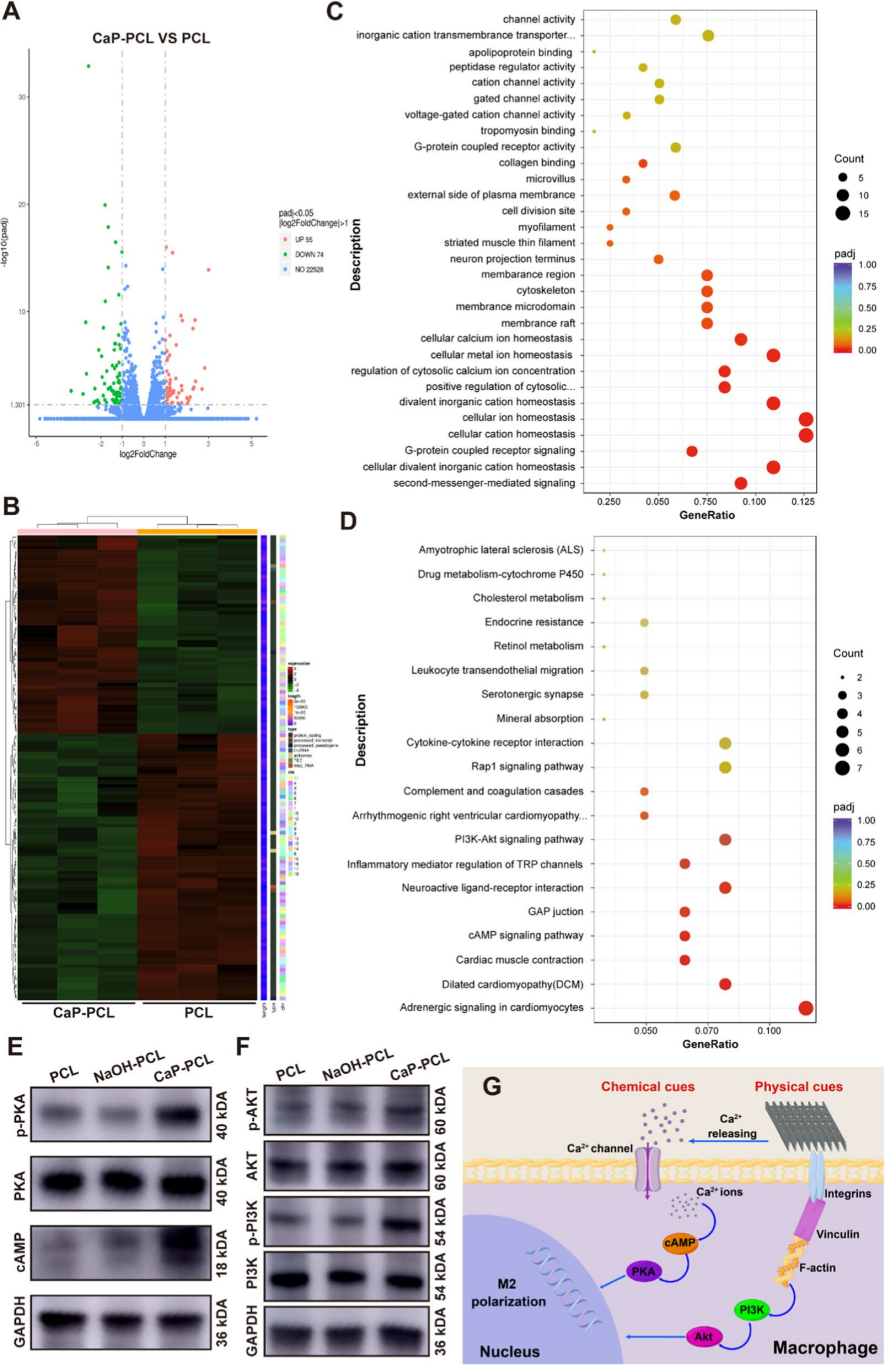
Mechanistic analysis of CaP-PCL induced macrophage polarization. **A**, **B** Volcano plot and heatmap of differently expressed genes of macrophages cultured on PCL and CaP-PCL scaffolds. **C** GO analysis of PCL versus CaP-PCL samples. **D** KEGG analysis of PCL in comparison with CaP-PCL samples. **E** The protein expressions of key markers in cAMP signaling pathway. **F** The protein expressions of key markers in PI3K-Akt signaling pathway. **G** Scheme illustration of the mechanism of CaP-PCL induced macrophage polarization. n = 3

To investigate how CaP-PCL scaffolds influenced macrophage polarization, a transcriptomic analysis was conducted on macrophages cultured on various scaffolds. According to the volcano plots, there were 55 genes that showed up-regulation and 74 genes that showed down-regulation in the PCL group comparison with the CaP-PCL group (Fig. 5A, B). Subsequently, the differentially expressed genes (DEGs) were utilized to perform Gene Ontology (GO) database analysis. The findings revealed that the genes were enriched in cellular calcium ion homeostasis and cytoskeleton, suggesting that CaP-PCL induced macrophage polarization might be associated with the regulation of macrophage morphological changes and calcium ion alterations (Fig. 5C). Recent studies have shown that macrophage polarization can be effectively regulated through the use of physical and chemical cues provided by biomaterials [14]. Physical cues such as surface wettability and roughness have been found to stimulate the up-regulation of integrins and vinculins, leading to the formation of focal adhesions and rearrangement of the cytoskeleton, ultimately regulating macrophage polarization [58, 61]. Additionally, chemical cues in the form of organic or inorganic substances of biomaterials have been found to act as stimuli or messengers to regulate macrophage polarization-related pathways [6, 43]. Based on the results of GO enrichment (Fig. 5C), it could be inferred that CaP-PCL induced macrophage polarization was due to the synergistic effect of both chemical and physical cues. Next, the Kyoto Encyclopedia of Genes and Genomes (KEGG) was conducted to investigate the signaling pathways that might be influenced by the chemical and physical cues of CaP-PCL scaffold. The results presented in Fig. 5D demonstrated enrichment of the PI3K-AKT and cAMP signaling pathways, which was subsequently confirmed through western blot analysis (Fig. 5E, F). Previous study has found that the hydrophilicity of titanium surface stimulates the PI3K-AKT signaling pathway via integrin β1, resulting in modulation of the macrophage response [58]. This suggested that the physical properties of scaffolds could activate the PI3K-AKT signaling pathway. In our study, transcriptome analysis revealed a correlation between the PI3K-AKT signaling pathway and macrophage polarization. In addition, it has been reported that Ca^2+^ is capable of activating the cAMP signaling pathway, which has been found to have a close association with macrophage polarization [62]. Based on the results, a potential mechanism for how physical and chemical cues of CaP-PCL scaffold improved M2 polarization of macrophages was presented in Fig. 5G. First, the physical cues (surface wettability and roughness) could trigger the assembly of integrin and vinculin, resulting in the formation of focal adhesions. These focal adhesions could act as cellular mechano-sensors, triggering the activation of the PI3K-AKT pathway. Second, inorganic messenger Ca^2+^ derived from CaP coating could promote the M2 polarization of macrophages by activating the cAMP pathway. Overall, the CaP-PCL scaffold displayed excellent immunomodulation properties due to the combined effect of physical and chemical cues.

### Evaluation of the macrophage polarization in a subcutaneous implantation model

**Fig. 6:**
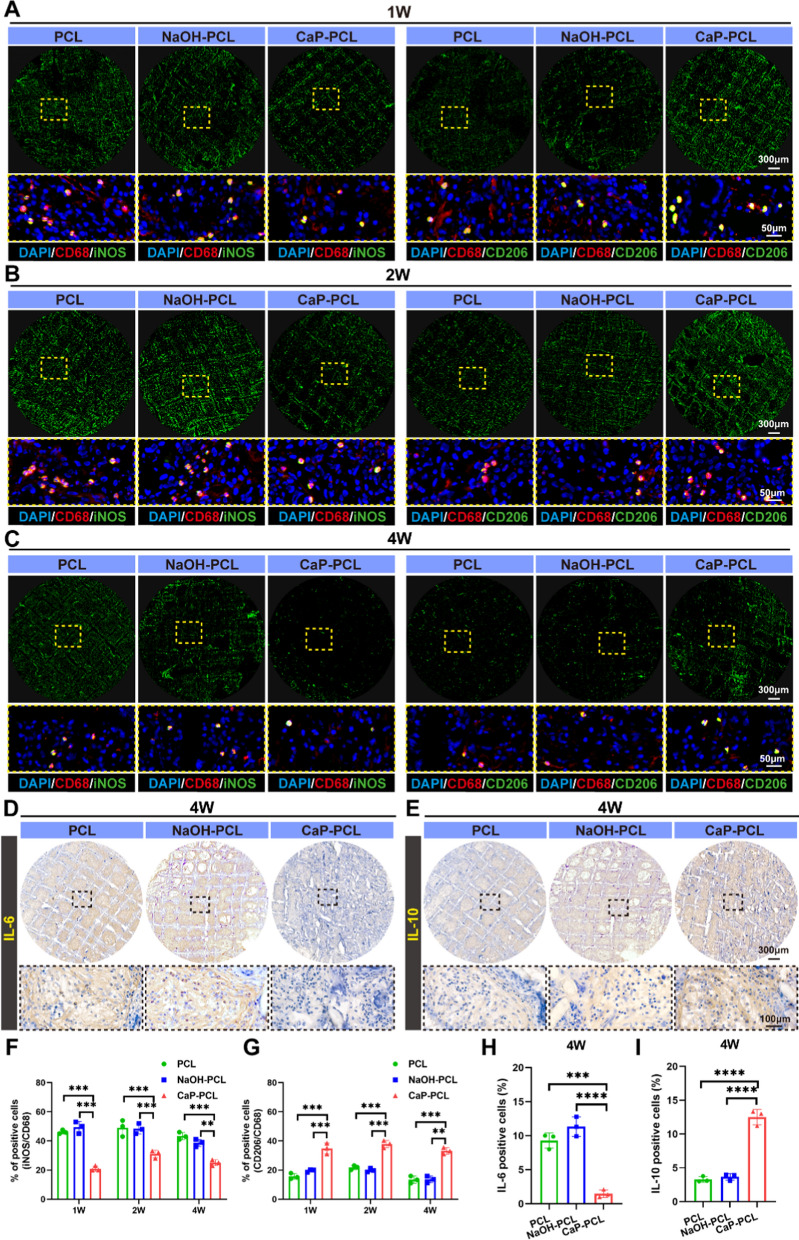
Evaluation of the macrophage polarization in a subcutaneous implantation model. **A**–**C** Immunofluorescent images of sections in different groups after subcutaneous implantation for 1, 2 and 4 weeks: red (M0 marker: CD68), green (M1 marker: iNOS and M2 marker: CD206). **D**, **E** Immunochemistry images of sections in different groups after subcutaneous implantation for 4 weeks. **F**, **G** The proportion of iNOS/CD68 and CD206/CD68 positive cells at different groups from 1 to 4 weeks. **H**, **I** The proportion of IL-6 and IL-10 positive cells after subcutaneous implantation for 4 weeks. n = 9. **P < 0.01, ***P < 0.001 and ****P < 0.0001

Previous studies have demonstrated that M1 macrophages secrete a diverse range of inflammatory factors, thereby augmenting the inflammatory response [63]. Conversely, M2 macrophages release anti-inflammatory factors, which facilitate tissue repair and remodeling [64]. Therefore, efficient and timely switching of macrophage phenotype from M1 to M2 is crucial for tissue healing. To evaluate the effect of CaP-PCL scaffold on macrophage polarization, a subcutaneous implantation was performed in rats. The images of HE staining revealed scattered distribution of immune cells, including monocytes and macrophages, in all three types of scaffolds (Additional file 1: Fig. S7A). The number of cells in all scaffolds exhibited a similar trend, showing a slight decrease over time. Furthermore, there was no significant difference in the cell count among the scaffolds on day 7, 14, and 28 (Additional file 1: Fig. S7B). Interestingly, a significant presence of blood vessels was observed within the CaP-PCL scaffold at day 28 (Additional file 1: Fig. S7A). To evaluate the phenotypic transformation of macrophages within the scaffolds, immunofluorescent staining was performed. The total number of macrophages was assessed by labeling CD68, while the switching from M1 to M2 phenotype was determined by labeling cells positive for iNOS and CD206, respectively. Consistent with the results of HE staining, the immunofluorescence images demonstrated a slight decrease in the proportion of macrophages in all scaffolds from day 7 to 28 (Fig. 6A–C). However, quantitative analysis demonstrated a significantly higher proportion of M2 macrophages and a lower proportion of M1 macrophages in the CaP-PCL scaffold group compared to the other two groups on day 7, 14, and 28 (Fig. 6F, G). Subsequently, the pro-healing cytokines (IL-10) and pro-inflammatory cytokines (IL-6) within various scaffolds were evaluated using immunochemistry (Fig. 6D, E, Additional file 1: Fig. S8). The results showed a significant decrease in the deposition of IL-6 in the CaP-PCL group compared to the other two groups on day 7, 14, and 28 (Fig. 6H, Additional file 1: Fig. S8). In contrast, there was a noticeable increase in the deposition of IL-10 in the CaP-PCL group compared to the other two groups at different time points (Fig. 6I, Additional file 1: Fig. S8). These results, along with the increase in phenotypic conversion of M1 to M2, provide further confirmation of the strong immunomodulatory activity of CaP-PCL scaffold in vivo.

### Macrophage phenotypic switching in bone defect model

**Fig. 7:**
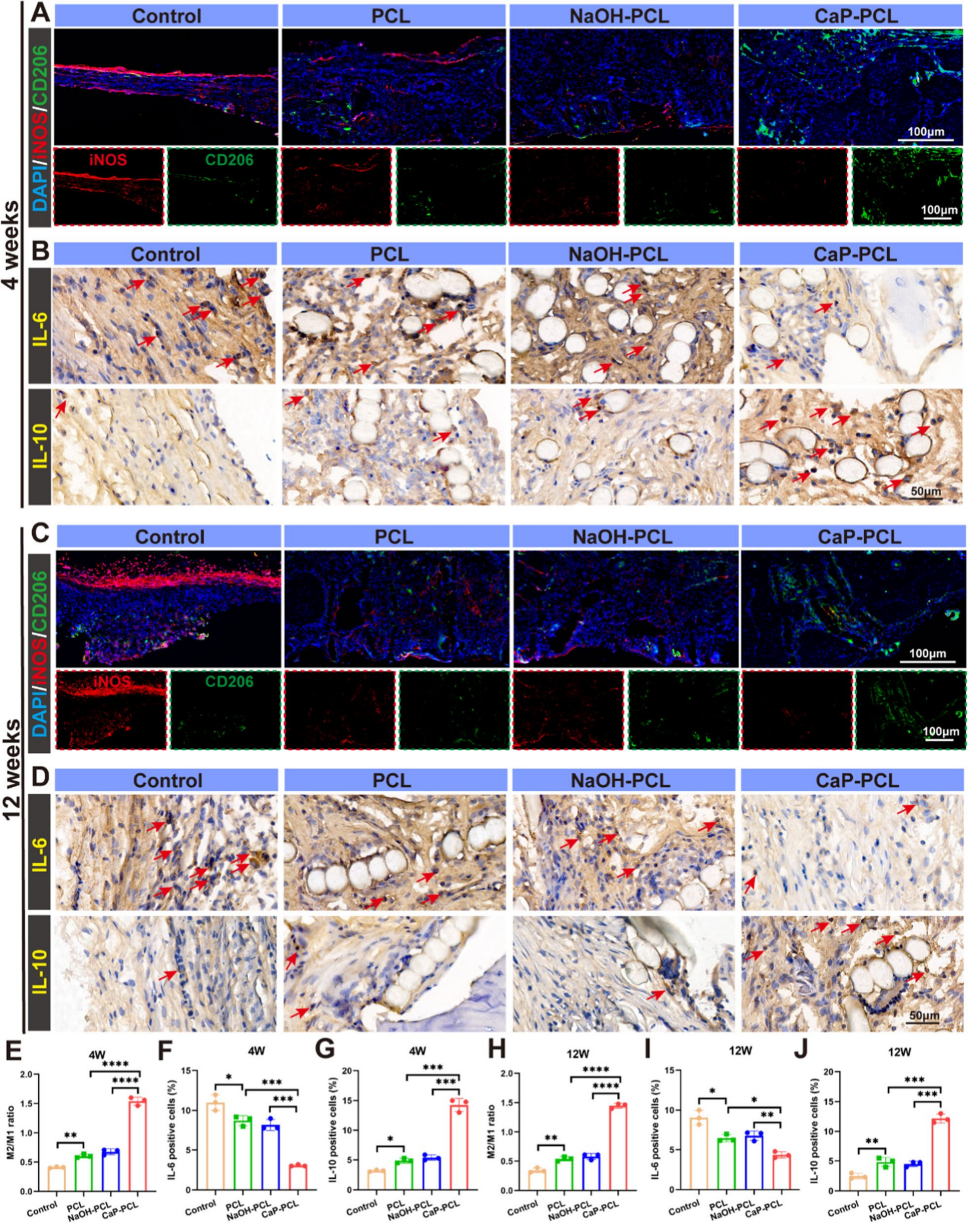
Macrophage phenotypic switching in bone defect model. **A** Immunofluorescent images of sections in different groups after implantation for 4 weeks: red (M1 marker: iNOS), green (M2 marker: CD206). **B** Immunochemistry images of sections in different groups after implantation for 4 weeks. **C** Immunofluorescent images of sections in different groups after implantation for 12 weeks. **D** Immunochemistry images of sections in different groups after implantation for 12 weeks. **E** The M2/M1 ratio of different groups after 4 weeks of implantation. **F**, **G** The proportion of IL-6 and IL-10 positive cells after 4 weeks of implantation. **H** The M2/M1 ratio of different groups after 12 weeks of implantation. I, J The proportion of IL-6 and IL-10 positive cells after 12 weeks of implantation. n = 6. *P < 0.05, **P < 0.01, ***P < 0.001 and ****P < 0.0001

Macrophages play a crucial role in osteo-immunomodulation because of their direct involvement in the initial inflammatory process of new bone formation [65]. The transition to the M2 phenotype has been demonstrated as crucial factor for the successful integration of implants and bone [66]. The immunomodulatory activity of CaP-PCL in modulating macrophage M1/M2 polarization had been verified in a subcutaneous implantation model. However, the immunomodulatory activity of CaP-PCL scaffolds in bone defect models remained unclear. Therefore, we conducted an investigation to assess the immunomodulatory activity of the CaP-PCL scaffold using a bone defect model. The switching from M1 to M2 phenotype was determined by labeling cells positive for CD86 and CD206, respectively. The results demonstrated a significantly larger number of M2 macrophages and a smaller number of M1 macrophages in the CaP-PCL scaffold group compared to the other two groups at 4 and 12 weeks after implantation (Fig. 7A, C). Additionally. the CaP-PCL scaffold demonstrated a noticeably higher M2/M1 ratio in comparison to the other two scaffolds at both time points (Fig. 7E, H).

Subsequently, the immunochemical results revealed a significant decrease in the deposition of the pro-inflammatory cytokine IL-4 (Fig. 7B, D, F, G) and a significant increase in the deposition of the anti-inflammatory cytokine IL-10 in the CaP-PCL group compared to the other two groups after 4 and 12 weeks of implantation (Fig. 7B, D, I, J). These results demonstrated that CaP-PCL had a strong stimulatory effect on macrophage polarization towards the M2 type in bone defect. This suggested that CaP-PCL has an osteoimmunomodulatory effect on bone regeneration, indicating its potential in promoting bone healing.

### Bone regeneration of CaP-PCL scaffold in vivo

**Fig. 8:**
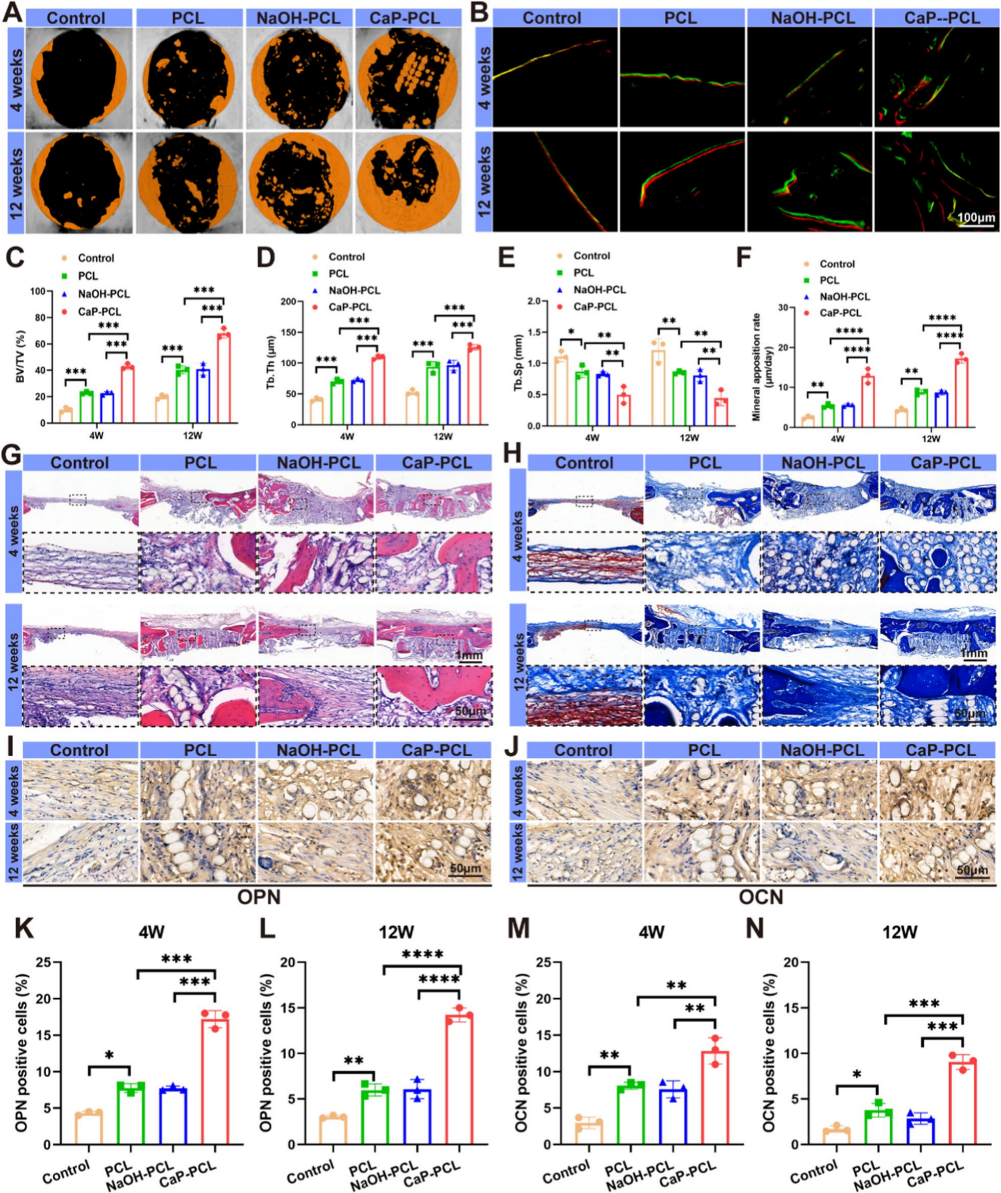
Bone regeneration of different scaffolds in vivo. **A** 3D micro-CT images of different groups. **B** Calcein-Alizarin Red staining images of different groups. **C**–**E** The quantification results of BV/TV, Tb.Th and Tb.Sp at different groups. **F** The quantification of mineral apposition rate at different groups. **G** HE staining of different sections. **H** Masson staining of different sections. **I**, **J** Immunochemistry images of OPN and OCN at different groups after implantation for 4 and 12 weeks. **K**, **L** The proportion of OPN positive cells at different groups after implantation for 4 and 12 weeks. **M**, **N** The proportion of OCN positive cells at different groups after implantation for 4 and 12 weeks. n = 6. *P < 0.05, **P < 0.01, ***P < 0.001 and ****P < 0.0001

The bone regeneration has been found to depend on a favorable immune microenvironment [67]. This microenvironment plays a crucial role in promoting osteogenesis differentiation and new bone formation around the implants [68]. Osteo-immunomodulation refers to the ability of implantable biomaterials to modulate the osteoimmune environment, thereby regulating the formation of new bone [69]. After confirming the optimal immunoactivity of the CaP-PCL scaffold in regulating M1/M2 polarization of macrophage both in vitro and in vivo, as well as its significant enhancement of osteogenesis in vitro, we proceeded to evaluate the new bone formation surrounding the scaffold. In order to evaluate the impact of bone regeneration in a bone defect model, 3D micro-CT reconstruction and histological analysis were performed. The 3D micro-CT images revealed that there was no substantial formation of new bone in the control group following a 12-week implantation period. This suggested that the bone exhibited limited regenerative capacity to address such a critical size defect (Fig. 8A). As expected, CaP-PCL scaffolds showed a higher amount of newly formed bone compared to the other two scaffolds at both the 4 and 12-week time points. Quantitative analysis also supported these findings, indicating that the CaP-PCL group had the highest percentage of bone volume to tissue volume (BV/TV) and displayed superior trabecular structural characteristics of the newly formed bone, including trabecular thickness (Tb.Th) and trabecular separation (Tb. Sp) (Fig. 8C–E). Fluorescent double labeling of Alizarin Red (red) and Calcein (green) was conducted to assess new bone formation. The CaP-PCL scaffold exhibited a greater separation between the two fluorescent signals (Fig. 8B). Additionally, quantitative analysis revealed that the CaP-PCL scaffold had a higher mineral content (MAR) compared to the other two scaffolds (Fig. 8F). In the context of osteointegration, it is essential for the degradation rate of the scaffold to match the growth rate of new bone. The micro-CT analysis revealed an increase in degradation rates of all the scaffolds over time. Furthermore, after 3 months of implantation, approximately 58% of scaffold degraded in CaP-PCL group (Additional file 1: Fig. S9). For future study, more effective ways need to find to enhance the degradation PCL in order to achieve better osteointegration.

After a period of 4 weeks following implantation, the results of HE staining demonstrated that the control group displayed thin fibrous tissue at the site of the defect. In contrast, the experimental groups exhibited varying degrees of capability in promoting new bone regeneration, particularly the CaP-PCL group. After a period of 12 weeks, a higher level of bone formation was observed in CaP-PCL scaffolds, leading to the development of a bony bridge at the defect sites (Fig. 8G). To evaluate the maturity of the newly formed bone tissue, Masson's staining was conducted to assess collagen deposition. The findings revealed that the CaP-PCL scaffolds exhibited the highest level of collagen deposition compared to the other two scaffolds (Fig. 8H). Similarly, Van Gieson staining also confirmed the presence of more bone calluses in CaP-PCL scaffolds, which aligned with the results obtained from H&E and Masson’s staining (Additional file 1: Fig. S10). Immunohistochemistry (IHC) analysis demonstrated increased levels of OPN and OCN expression in the CaP-PCL group at both 4- and 12-weeks post-implantation (Fig. 8I, J). Quantitative analysis revealed that the positive cells of the CaP-PCL group was significantly greater in comparison to the other two groups (Fig. 8K–N). The results presented above demonstrated that the CaP-PCL scaffold effectively modulated the immune microenvironment, thereby promoting new bone regeneration within the bone defect area.

## Conclusions

While our study successfully detected the release of calcium ions in vitro, it was important to note that detecting calcium ion release at bone defects posed a significant challenge. Calcium ions exist in various forms and can interact with other molecules, making them difficult to quantify. Moreover, the concentration of calcium ions in bone defect is influenced by various factors such as tissue damage severity, intracellular calcium ion release, and metabolism. Therefore, it is difficult to accurately detect calcium ion release in vivo. In future study, fluorescence imaging or radiolabeling may be employed to visualize or track the release of calcium ions in real-time, thus providing valuable insights into their spatial and temporal distribution.

In summary, calcium phosphate (CaP) coating was performed on a PCL scaffold with the aim of enhancing its osteoimmunomodulatory effect. The study revealed that the CaP coated PCL scaffold exhibited a rougher surface topography and higher hydrophilicity in comparison to the bare PCL scaffold. Besides, the release of Ca^2+^ from the CaP coating and the surface morphology of the coatings might be two important factors in regulating the transition of macrophages from M1 to M2 phenotypes. The osteoimmune microenvironment induced by CaP coated PCL scaffolds not only enhanced the osteogenic differentiation of BMSC in vitro but also contributed to the bone regeneration in vivo. Overall, the utilization of CaP coating on PCL scaffolds can be employed to control the phenotypic switching of macrophages, thereby creating a beneficial immunomodulatory microenvironment to promote bone regeneration. This study offers novel insights into the developing tissue-engineered implants with immune activity.

### Supplementary Information

**Additional file 1: Table S1.** Details of primary and secondary antibodies. **Table S2.** The premiers used in this study. **Table S3.** Quantitative analysis of EDS elemental mapping. **Figure S1.** The zeta potential of different scaffolds. n = 3. *P < 0.05 and **P < 0.01. **Figure S2.** The mechanical results of different scaffolds. **A** The representative images of tensile test. **B**, **C** The tensile Young’s modulus and tensile strength of different scaffolds. **D** The representative images of compressive test. **B**, **C** The compressive Young’s modulus and compressive strength of different scaffolds. n = 3. *P < 0.05, **P < 0.01 and ***P < 0.001. **Figure S3.** The ex vivo calcium release of CaP coating from CaP-PCL scaffold. n = 3. **Figure S4.** The results of in vitro degradability of scaffolds. n = 3. **Figure S5.** The mRNA expressions and immunofluorescent staining of osteogenic differentiation markers. **A** The mRNA expressions of osteogenic differentiation markers in BMSC after culture for 7 days. **B** The mRNA expressions of osteogenic differentiation markers in BMSC after culture for 14 days. **C**–**E** Immunofluorescent images of ALP, OPN and RUNX2 in BMSC. **F** The area fraction of ALP, OPN and RUNX2. n = 3. *P < 0.05, **P < 0.01 and ***P < 0.001. **Figure S6.** In vitro evaluation of osteoinductive and osteoimmunomodulatory effects of different scaffolds. **A** Two types of culture conditions were employed for BMSCs. **B**, **D** ALP staining of BMSCs and the quantification results. **C**, **E** ARS staining of BMSCs and the quantification results. **F** The protein expressions of osteogenic differentiation markers. n = 3. **P < 0.01 and ***P < 0.001. **Figure S7.** HE staining of sections in different groups. **A** HE staining of sections in different groups after subcutaneous implantation for 1, 2 and 4 weeks. **B** The proportion of inflammation related cells in different scaffolds, n = 9. **Figure S8.** Immunochemistry images of sections in different groups. **A**, **B**, **E**, **F** Immunochemistry staining of IL-6 and IL-10 in different groups after subcutaneous implantation for 1 week (**A**, **B**) and 2 weeks (**E**, **F**). **C**, **D**, **G**, **H** The proportion of IL-6 and IL-10 positive cells in different groups after subcutaneous implantation for 1 week (**C**, **D**) and 2 weeks (**G**, **H**). n = 6. ***P < 0.01 and ****P < 0.0001. **Figure S9.** The results of in vivo degradability among three different scaffolds. **A** 3D micro-CT images of different scaffolds after 4 weeks and 12 weeks of implantation. **B** The degradation rate of different scaffolds after 4 weeks of implantation. **C** The degradation rate of different scaffolds after 12 weeks of implantation. n = 6, ***P < 0.001. **Figure S10.** Van Gieson staining of different groups after implantation for 4 and 12 weeks.

## Data Availability

The data are available from the corresponding author upon reasonable request.
