# Supplementary material for: Calcium phosphate coating enhances osteointegration of melt electrowritten scaffold by regulating macrophage polarization
Source: J Nanobiotechnology. 2024 Jan 31;22:47. doi: 10.1186/s12951-024-02310-0 (PMC10829397; doi:10.1186/s12951-024-02310-0)
Supplement: Supplementary file 1 — Additional file 1: Table S1. Details of primary and secondary antibodies. Table S2. The premiers used in this study. Table S3. Quantitative analysis of EDS elemental mapping. Figure S1. The zeta potential of different scaffolds. n = 3. *P < 0.05 and **P < 0.01. Figure S2. The mechanical results of different scaffolds. A The representative images of tensile test. B, C The tensile Young’s modulus and tensile strength of different scaffolds. D The representative images of compressive test. B, C The compressive Young’s modulus and compressive strength of different scaffolds. n = 3. *P < 0.05, **P < 0.01 and ***P < 0.001. Figure S3. The ex vivo calcium release of CaP coating from CaP-PCL scaffold. n = 3. Figure S4. The results of in vitro degradability of scaffolds. n = 3. Figure S5. The mRNA expressions and immunofluorescent staining of osteogenic differentiation markers. A The mRNA expressions of osteogenic differentiation markers in BMSC after culture for 7 days. B The mRNA expressions of osteogenic differentiation markers in BMSC after culture for 14 days. C–E Immunofluorescent images of ALP, OPN and RUNX2 in BMSC. F The area fraction of ALP, OPN and RUNX2. n = 3. *P < 0.05, **P < 0.01 and ***P < 0.001. Figure S6. In vitro evaluation of osteoinductive and osteoimmunomodulatory effects of different scaffolds. A Two types of culture conditions were employed for BMSCs. B, D ALP staining of BMSCs and the quantification results. C, E ARS staining of BMSCs and the quantification results. F The protein expressions of osteogenic differentiation markers. n = 3. **P < 0.01 and ***P < 0.001. Figure S7. HE staining of sections in different groups. A HE staining of sections in different groups after subcutaneous implantation for 1, 2 and 4 weeks. B The proportion of inflammation related cells in different scaffolds, n = 9. Figure S8. Immunochemistry images of sections in different groups. A, B, E, F Immunochemistry staining of IL-6 and IL-10 in different groups after subcutaneous [file 12951_2024_2310_MOESM1_ESM.docx]

**Additional file 1**

**Table S1: Details of primary and secondary antibodies.**

| Name | Dilution | Supplier | ­­Catalog no. |
| --- | --- | --- | --- |
| cAMP | 1:1000 | Abclonal | A15653 |
| PKA | 1:1000 | Affinity | AF7746 |
| p-PKA | 1:1000 | Affinity | AF7246 |
| PI3K | 1:1000 | Abmart | T40064 |
| p-PI3K | 1:1000 | Abmart | T40116 |
| Akt | 1:1000 | CST | 4691 |
| p-Akt | 1:1000 | CST | 4060 |
| ALP | 1:1000 | Santa Cruz | sc-365765 |
| OPN | 1:1000 | PTGCN | 22952-1-AP |
| Runx2 | 1:1000 | PTGCN | 20700-1-AP |
| CD86 | 1:1000 | PTGCN | 13395-1-AP |
| iNOS | 1:1000 | PTGCN | 22226-1-AP |
| CD206 | 1:1000 | PTGCN | 18704-1-AP |
| Arg-1 | 1:1000 | PTGCN | 16001-1-AP |
| GAPDH | 1:5000 | PTGCN | 11-7423-82 |
| HRP-Goat anti-rabbit IgG (H+L) | 1:5000 | PTGCN | SA00001-2 |

**Table S2: The premiers used in this study.**

| Gene | Forward | Reverse |
| --- | --- | --- |
| RUNX2 | ATGCTTCATTCGCCTCACAAAC | TAAATGACTCGGTTGGTCTCGG |
| ALP | GGAACAACCTGACTGACCCTTC | AGTCAAGGTGTCTTTCTGGGATG |
| OPN | GATTGGCAGTGATTTGCTTTTGC | AGATTCTGCTTCTGAGATGGGTC |
| TNF-α | GTTCCCAAATGGCCTCCC | GTGCTCCTCACCCACACCG |
| IL-6 | CTCTGGGAAATCGTGGAAAT | CCAGTTTGGTAGCATCCATC |
| IL-4 | GAGGATCAGCAGGGGCCAGTAC | AAGGCAGTCCGCAGCTCTAGG |
| IL-10 | CCCTTTGCTATGGTGTCCT | GTGGCCAGTTTGTTATTTAT |
| GAPDH | AAGGTCGGTGTGAACGGATTT | TGAGGTCAATGAAGGGGTCG |

| Atomic concentration | C | O | Ca | P | Na | Ca/P |
| --- | --- | --- | --- | --- | --- | --- |
| PCL | 76.52 | 23.48 | 0.00 | 0.00 | 0.00 |  |
| NaOH-PCL | 75.62 | 24.20 | 0.00 | 0.00 | 0.00 |  |
| CaP-PCL | 60.32 | 20.45 | 11.37 | 7.68 | 0.00 | 1.48 |

**Table S3: Quantitative analysis of EDS elemental mapping.**

**
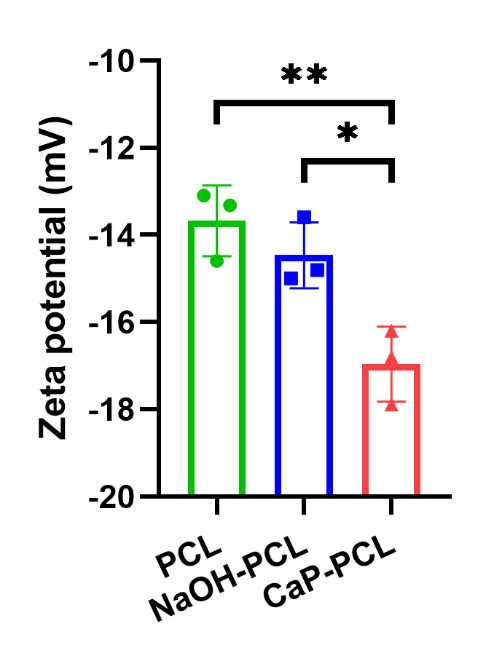
**

**Figure S1: The zeta potential of different scaffolds.** n = 3. **P*<0.05 and ***P*<0.01.

**
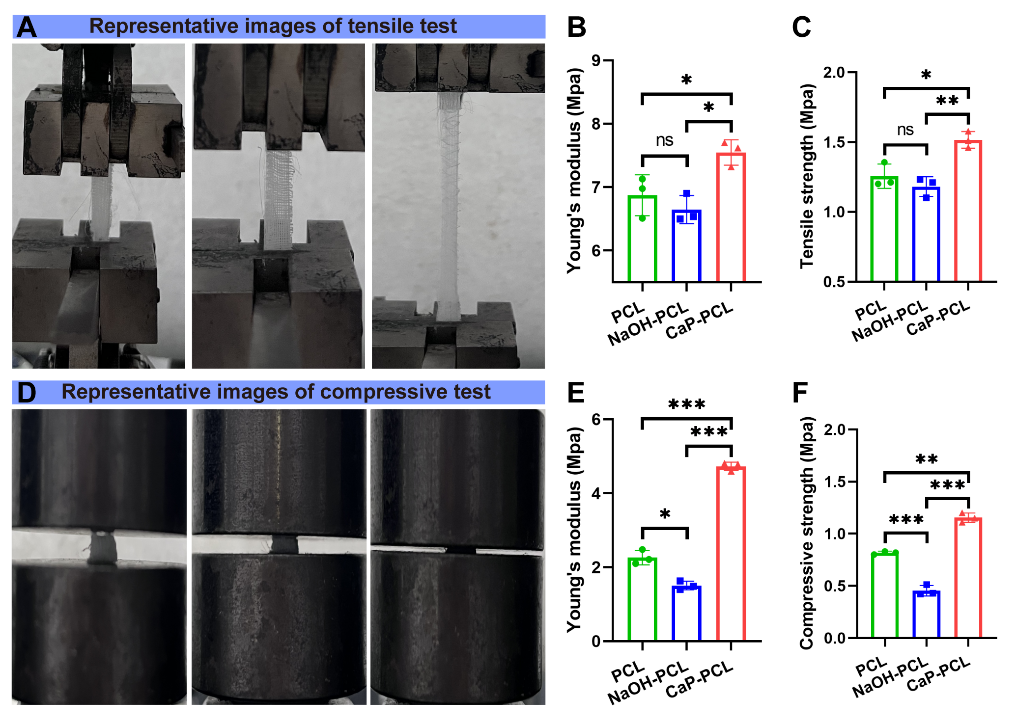
**

**Figure S2: The mechanical results of different scaffolds.** (A) The representative images of tensile test. (B, C) The tensile Young’s modulus and tensile strength of different scaffolds. (D) The representative images of compressive test. (B, C) The compressive Young’s modulus and compressive strength of different scaffolds. n = 3. **P*<0.05, ***P*<0.01 and ****P*<0.001.


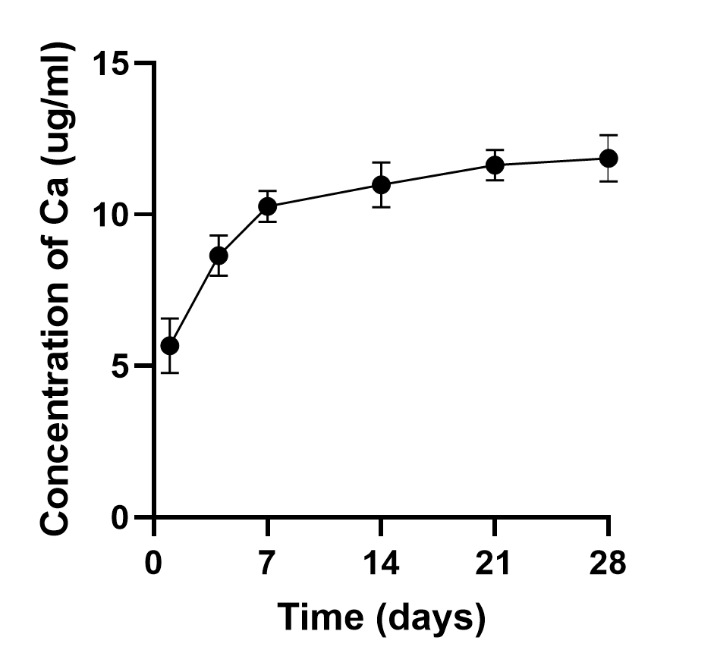


**Figure S3: The ex vivo calcium release of CaP coating from CaP-PCL scaffold.** n = 3.


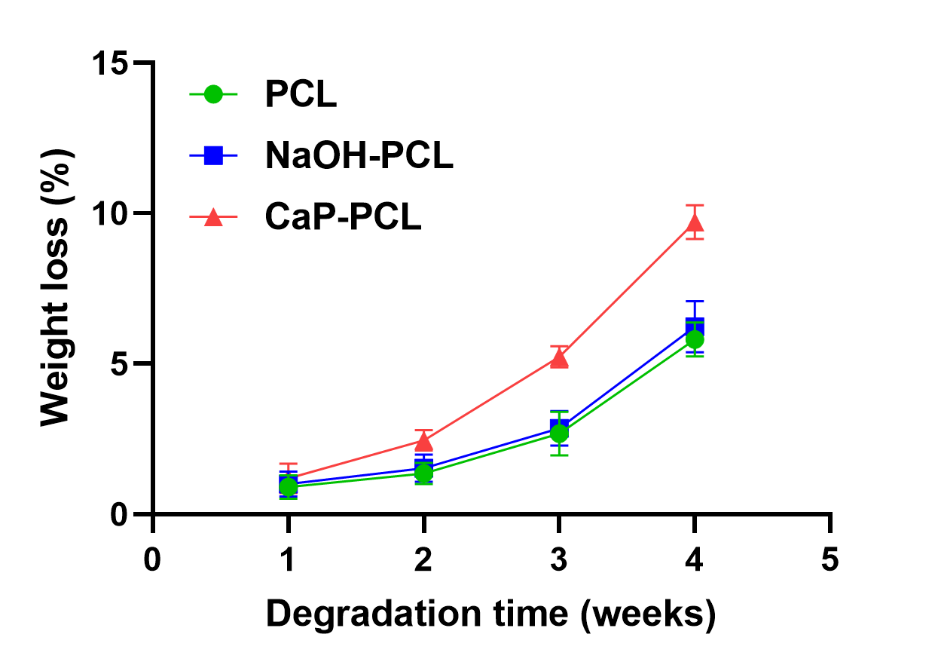


**Figure S4: The results of in vitro degradability of scaffolds.** n = 3.

**
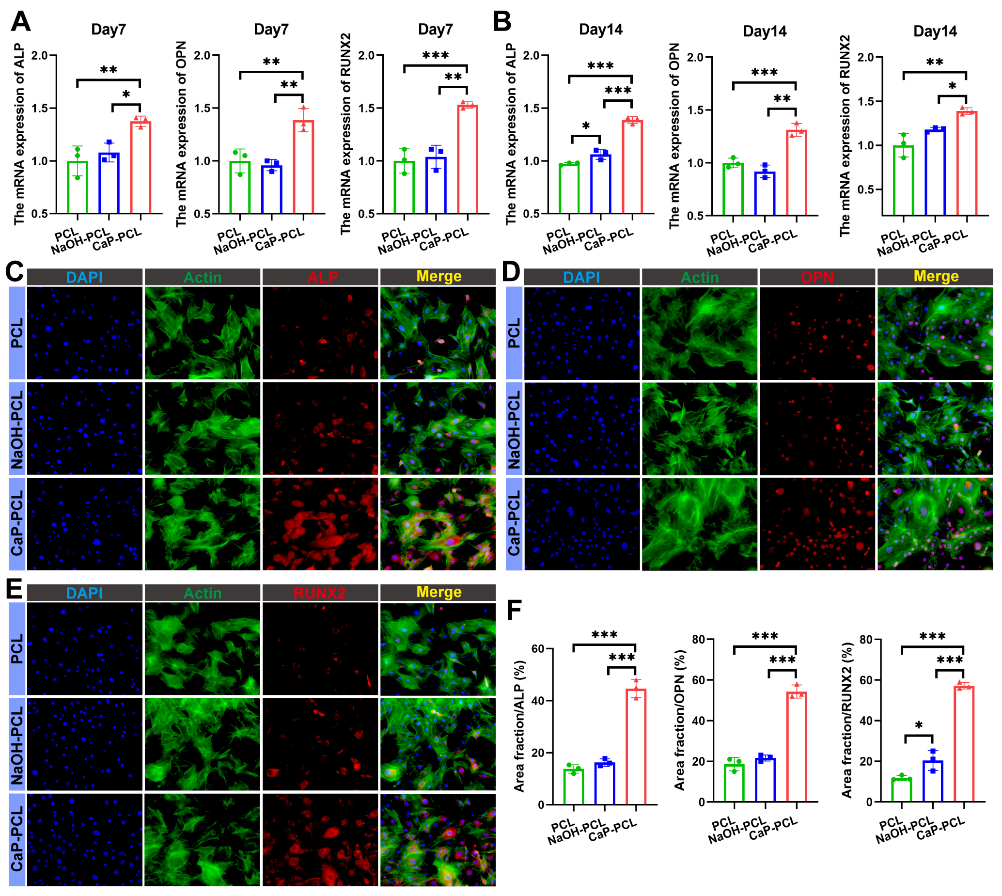
**

**Figure S5: The mRNA expressions and** **immunofluorescent staining of osteogenic differentiation markers.** (A) The mRNA expressions of osteogenic differentiation markers in BMSC after culture for 7 days. (B) The mRNA expressions of osteogenic differentiation markers in BMSC after culture for 14 days. (C, D, E) Immunofluorescent images of ALP, OPN and RUNX2 in BMSC. (F) The area fraction of ALP, OPN and RUNX2. n = 3. **P*<0.05, ***P*<0.01 and ****P*<0.001.


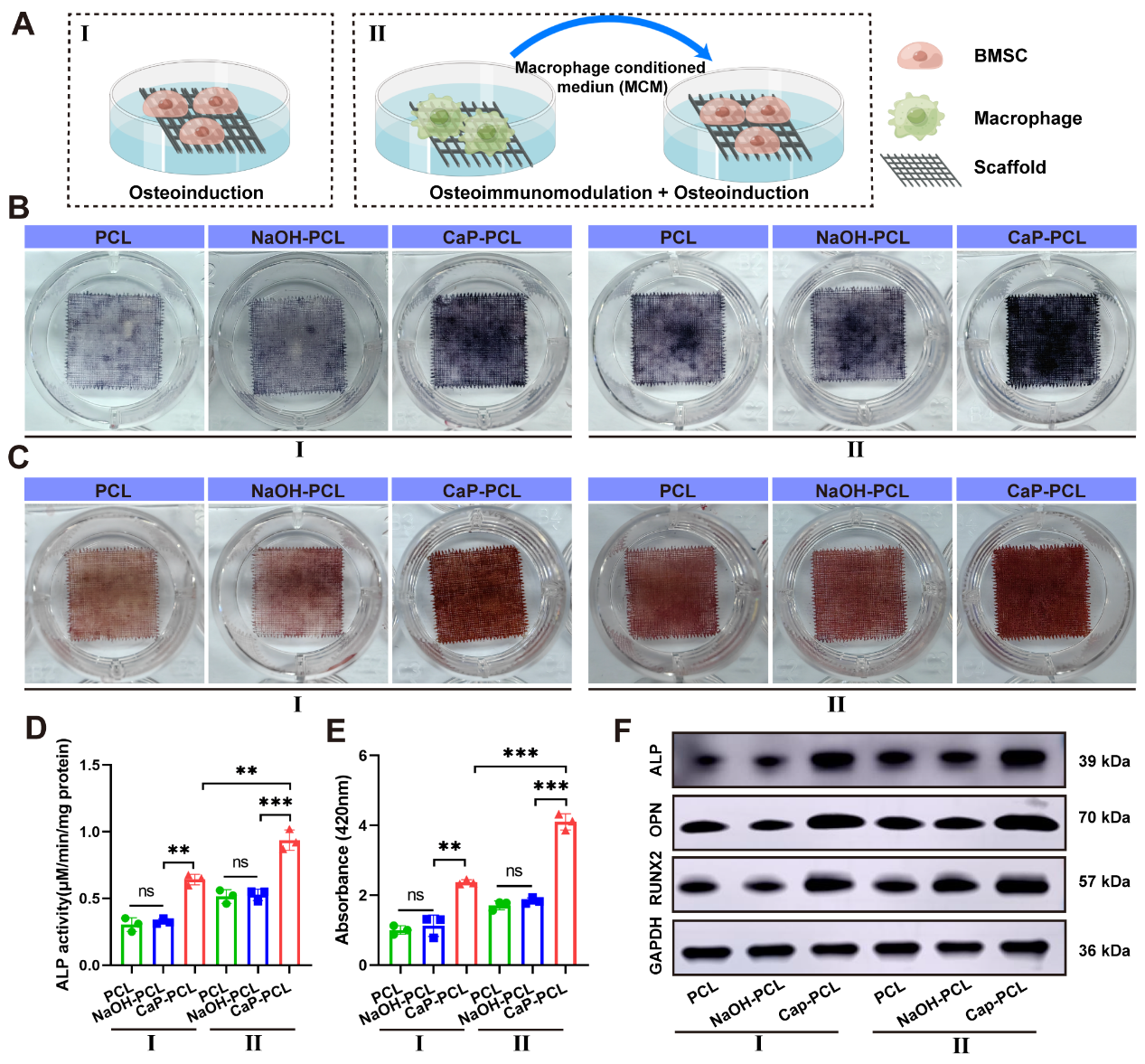


**Figure S6：In vitro evaluation of osteoinductive and osteoimmunomodulatory effects of different scaffolds.** (A) Two types of culture conditions were employed for BMSCs. (B, D) ALP staining of BMSCs and the quantification results. (C, E) ARS staining of BMSCs and the quantification results. (F) The protein expressions of osteogenic differentiation markers. n = 3. ***P*<0.01 and ****P*<0.001.

**
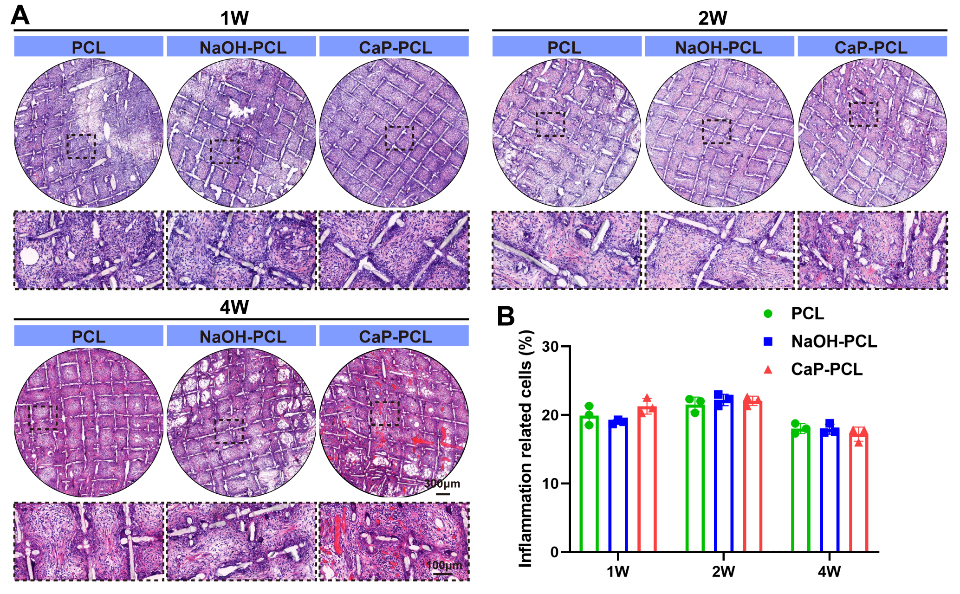
**

**Figure S7: HE staining of sections in different groups.** (A) HE staining of sections in different groups after subcutaneous implantation for 1, 2 and 4 weeks. (B) The proportion of inflammation related cells in different scaffolds. n = 9.

**
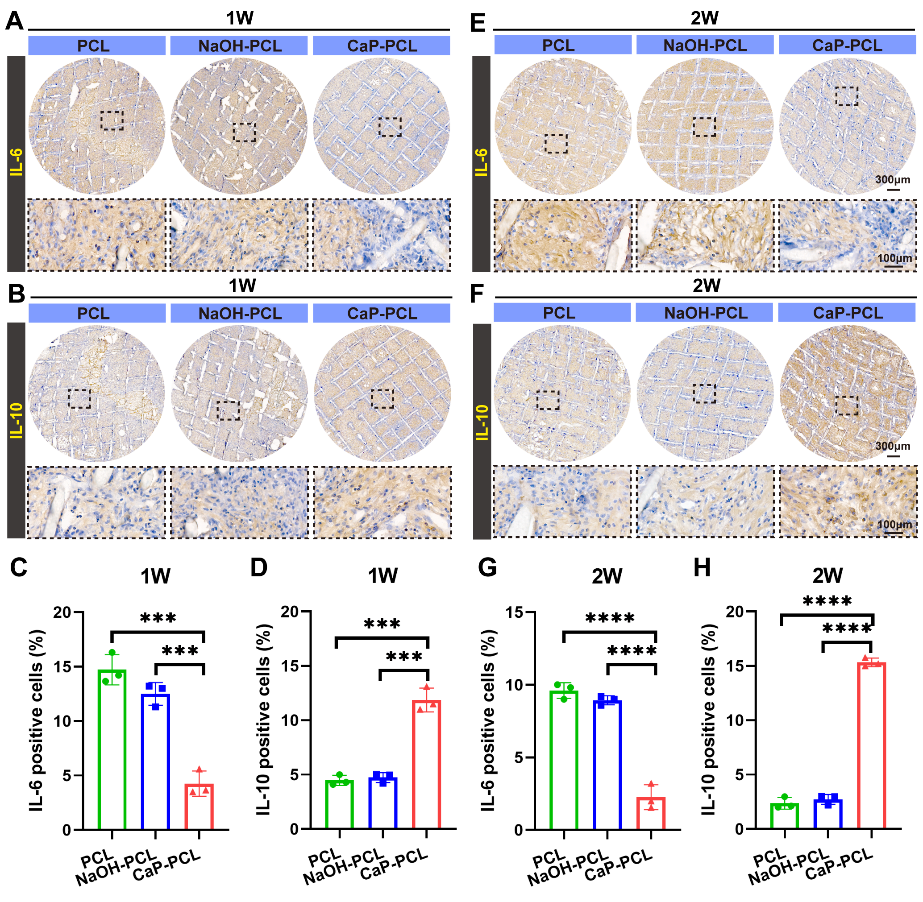
**

**Figure S8:** **Immunochemistry images of sections in different groups.** (A, B, E, F) Immunochemistry staining of IL-6 and IL-10 in different groups after subcutaneous implantation for 1 week (A, B) and 2 weeks (E, F). (C, D, G, H) The proportion of IL-6 and IL-10 positive cells in different groups after subcutaneous implantation for 1 week (C, D) and 2 weeks (G, H). n = 6. ****P*<0.01 and *****P*<0.0001.


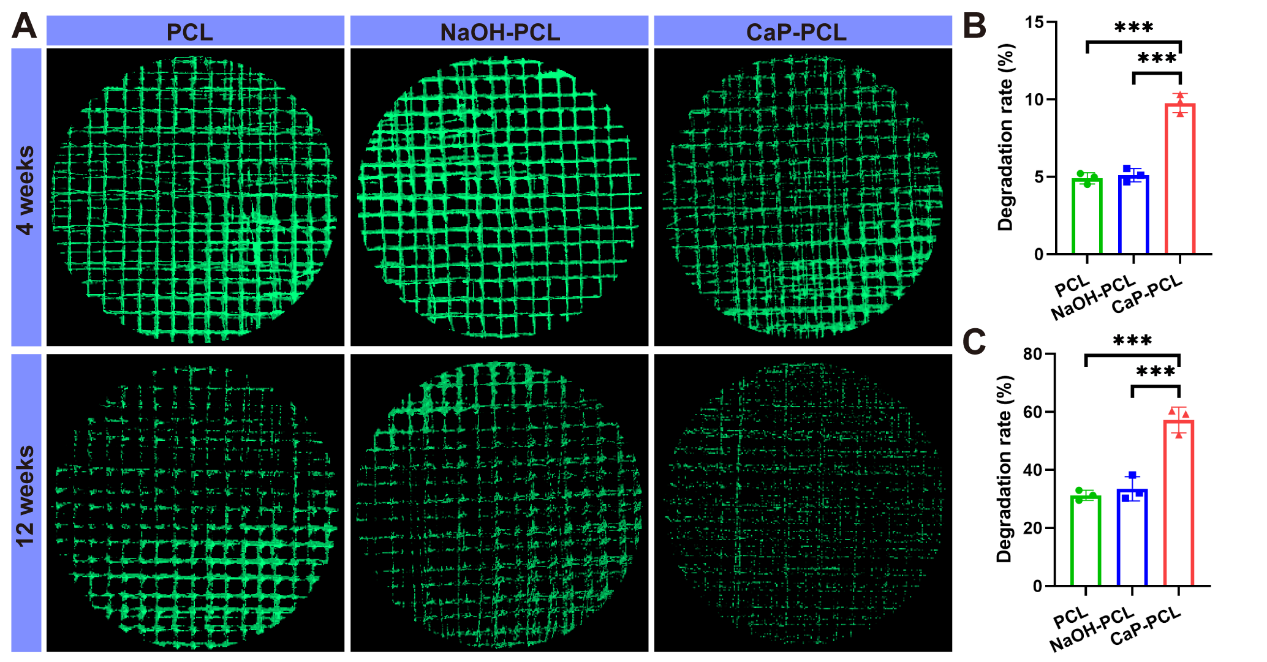


**Figure S9: The results of in vivo degradability among three different scaffolds**. (A) 3D micro-CT images of different scaffolds after 4 weeks and 12 weeks of implantation. (B) The degradation rate of different scaffolds after 4 weeks of implantation. (C) The degradation rate of different scaffolds after 12 weeks of implantation. n = 6, ****P*<0.001.

**
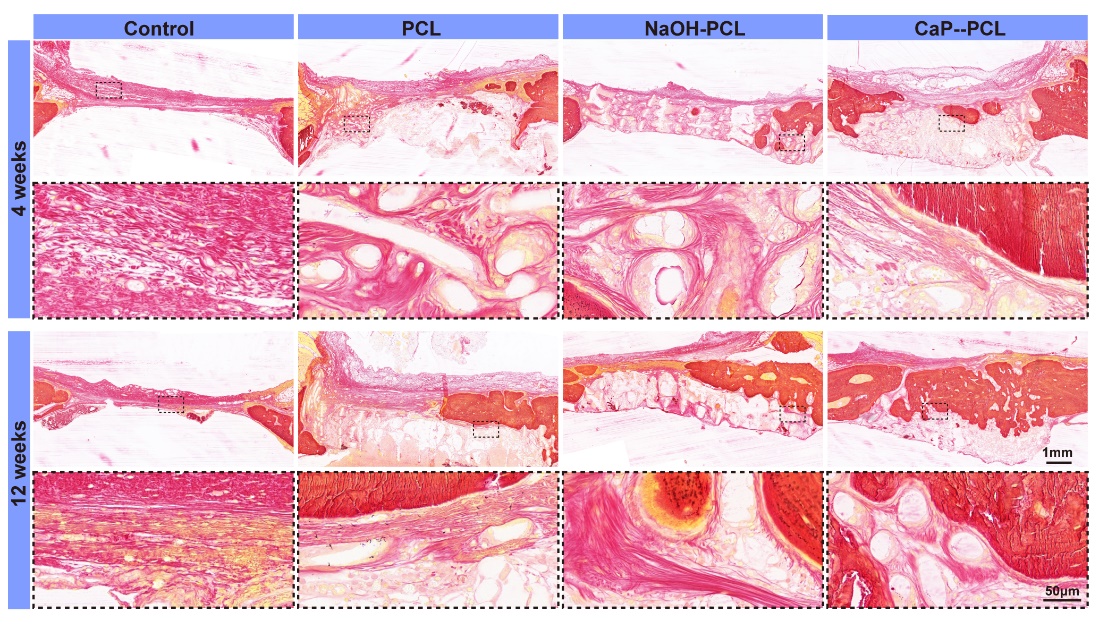
**

**Figure S10: Van Gieson staining of different groups after implantation for 4 and 12 weeks.**
